# SpaiNN: equivariant message passing for excited-state nonadiabatic molecular dynamics†

**DOI:** 10.1039/d4sc04164j

**Published:** 2024-09-02

**Authors:** Sascha Mausenberger, Carolin Müller, Alexandre Tkatchenko, Philipp Marquetand, Leticia González, Julia Westermayr

**Affiliations:** a Faculty of Chemistry, Institute of Theoretical Chemistry, University of Vienna Währinger Str. 17 1090 Vienna Austria; b Vienna Doctoral School in Chemistry (DosChem), University of Vienna Währinger Straße 42 1090 Vienna Austria; c Department Chemistry and Pharmacy, Computer-Chemistry-Center, Friedrich-Alexander-Universität Erlangen-Nürnberg Nägelsbachstraße 25 91052 Erlangen Germany carolin.cpc.mueller@fau.de; d Department of Physics and Materials Science, University of Luxembourg 162 A, Avenue de la Faïencerie L-1511 Luxembourg Luxembourg; e Wilhelm Ostwald Institute for Physical and Theoretical Chemistry, Leipzig University Linnéstraße 2 04103 Leipzig Germany Julia.westermayr@uni-leipzig.de; f Center for Scalable Data Analytics and Artificial Intelligence (ScaDS.AI) Dresden/Leipzig Germany

## Abstract

Excited-state molecular dynamics simulations are crucial for understanding processes like photosynthesis, vision, and radiation damage. However, the computational complexity of quantum chemical calculations restricts their scope. Machine learning offers a solution by delivering high-accuracy properties at lower computational costs. We present SpaiNN, an open-source Python software for ML-driven surface hopping nonadiabatic molecular dynamics simulations. SpaiNN combines the invariant and equivariant neural network architectures of SchNetPack with SHARC for surface hopping dynamics. Its modular design allows users to implement and adapt modules easily. We compare rotationally-invariant and equivariant representations in fitting potential energy surfaces of multiple electronic states and properties arising from the interaction of two electronic states. Simulations of the methyleneimmonium cation and various alkenes demonstrate the superior performance of equivariant SpaiNN models, improving accuracy, generalization, and efficiency in both training and inference.

## Abstract 2

SpaiNN employs SchNetPack to train electronic properties across various potential energy curves, including energies, gradients, and couplings, while integrating with SHARC for excited state molecular dynamics simulations.

## Graphical abstract

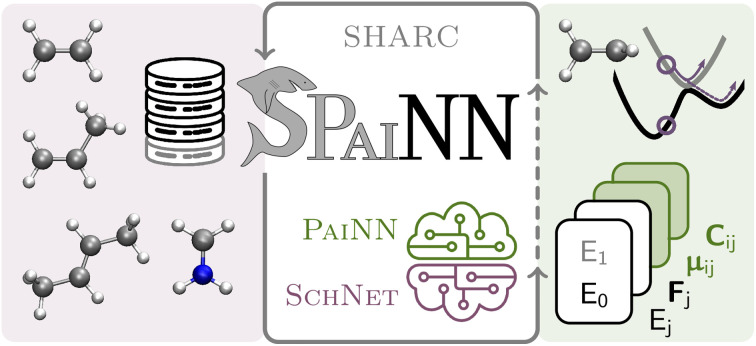

## 1 Introduction

Accurate non-adiabatic molecular dynamics (NAMD) simulations play a vital role across various research domains in photophysics and photochemistry. These simulations delve into the exploration of light-induced reactions, considering light as one of the most prevalent energy sources on Earth. They facilitate the exploration of diverse phenomena, including but not limited to,^1–3^ vision,^4,5^ or radiation damage.^6–8^ All these processes involve molecular reactions occurring after excitation to higher electronic states. By studying the subsequent nuclear dynamics, it is possible to uncover the intricate relationships between molecular structure and photochemical properties, paving the way towards the design of new photoactive molecules,^9^ including photocatalysts,^10^ photosensitive drugs,^11^ or photovoltaic materials.^12–14^ Despite the importance of these NAMD simulations, the prohibitive costs associated with accurate quantum chemistry calculations for photodynamics have limited the scope of excited-state simulations. These restrictions apply specifically to full quantum dynamics or semi-classical dynamics simulations, confining them to short time scales (usually several picoseconds) and small systems (typically a few dozen atoms).^9,15^ Typical semi-classical photodynamics simulations usually cover only a range of several picoseconds for a few dozen of atoms.^9,15^

With the advent of machine learning (ML) in theoretical chemistry, analytic representations of potential energy surfaces (PESs) and related properties have been developed.^16,17^ These approaches accelerate the simulations by decoupling the computational costs of electronic structure calculations from the dynamics simulations.^16^ While ground-state PES fitting has been pursued for over two decades and has enabled the simulation of molecular dynamics of systems with many thousands to millions of atoms with *ab initio* accuracy,^18–20^ the application of ML approaches to excited-state processes has appeared much later, with most developments occurring mainly in this century.^9,21–23^ As a consequence, there is significant methodological work to be done in accurately and meaningfully fitting the numerous excited-state PESs and associated properties.

Equivariant ML models have recently emerged for ground-state properties and have achieved notable success in fitting thereof. These models have demonstrated improved accuracy and efficiency compared to traditional ML models by successfully capturing the underlying symmetries of molecules and exploiting them to achieve accurate predictions even with limited training data.^24–28^ However, to the best of our knowledge, only one study has applied equivariant ML to describe excited state properties. Gómez-Bombarelli and co-workers developed a diabatic artificial neural network using the equivariant paiNN^29^ model, achieving a six-fold speedup in photodynamics simulations for azobenzene derivatives.^30^ Although their model demonstrated robustness and transferability within the chemical space of azobenzenes, it was not compared to the performance of invariant approaches, such as PyRAI^2^MD^31,32^ and SchNarc.^33^ The latter is based on SchNet, the invariant precursor to paiNN used in the aforementioned study.^30^ This omission makes it unclear whether equivariant models have an advantage over invariant models for excited states.

Nonetheless, It is expected that equivariant models will play a crucial role in enabling robust training and accurate prediction of vectorial properties,^29^ such as nonadiabatic couplings (NACs) and transition dipoles in NAMD simulations. Unlike paiNN, SchNet is rotationally invariant and cannot directly predict vectorial properties.^29,34^ Instead, SchNet predicts a virtual property, from which the equivariant property is derived by taking its derivative with respect to nuclear coordinates. Although SchNarc can predict NACs using this approach,^33,35^paiNN's equivariant design would allow for direct prediction of vectorial properties.

To address this gap, we developed SpaiNN, an open-source Python tool for ML-accelerated photodynamics simulations that facilitates the use of both invariant and equivariant architectures, allowing for a direct comparison and evaluation of the benefits of equivariance. SpaiNN integrates the NAMD program SHARC 3.0 (Surface Hopping Including ARbitrary Couplings)^36,37^ with the neural network potentials of SchNetPack 2.0.^38^ (including SchNet^34^ and paiNN modules). This interface allows SpaiNN to facilitate ML-based surface hopping NAMD, leveraging SchNetPack 2.0's inherent strengths, such as an improved data pipeline and support for both invariant (SchNet) and equivariant (paiNN) neural networks.^38^

We demonstrate the enhanced accuracy of SpaiNN models based on the paiNN compared to SchNet architecture across various aspects, including PESs, which encompass energies, forces and NACs. Additionally, we highlight the improved computational efficiency of SpaiNN(paiNN) with respect to SpaiNN(SchNet) models for both training and predictions. We showcase the performance of SpaiNN for a selection of alkenes and the methyleneimmonium cation by accurately predicting energies, forces, and NACs. These molecules are known to undergo rotation around the double bond upon photoexcitation. Furthermore, we illustrate the acceleration of NAMD simulations for the methyleneimmonium cation using SpaiNN models.

## 2 SpaiNN architecture

**Fig. 1:**
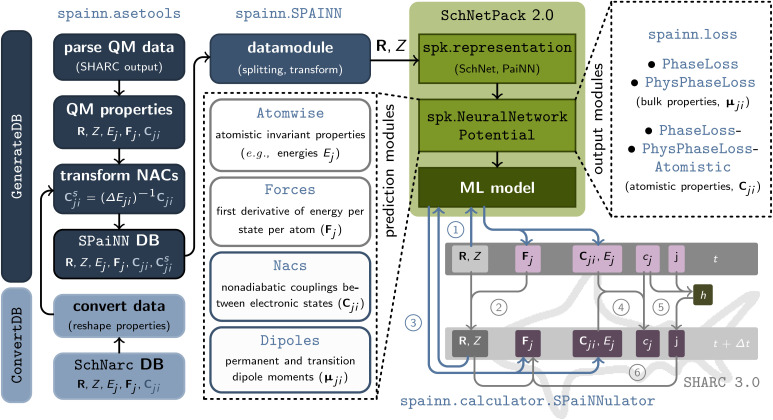
Overview of the various modules implemented in SpaiNN: the asetools module creates and manages datasets of Cartesian coordinates (**R**) and atomic charges (*Z*) as well as energies (*E*_*j*_), and forces (**F**_*j*_) across different electronic states, along with their coupling properties (**C**_*ji*_), by parsing SHARC output files (*GenerateDB*) or converting existing ASE databases (*ConvertDB*) into the required format (*cf.* Table S2†). Data handling is performed by an adapted AtomsDataModule class from SchNetPack 2.0 (ref. 38) (SpaiNN), which facilitates data splitting, scaling, and transformation for multiple electronic states. The neural network potentials for training electronic properties of various electronic states utilize standard SchNetPack 2.0 modules (*e.g.*, representation and neural network potential modules), with SpaiNN providing specialized prediction modules (*model*) for atomistic invariant properties (*Atomwise*), forces as energy derivatives (*Forces*), non-adiabatic couplings (*Nacs*), and dipole moments (*Dipoles*). SpaiNN also includes loss functions, output modules (*loss*), for phase-free training of properties arising from interactions between electronic states (**C**_*ji*_), using phase vectors (*PhysPhaseLoss*, *PhysPhaseLossAtomistic*) or ±1 multiplication (*PhaseLoss*, *PhaseLossAtomistic*) for on-the-fly phase correction during training. Additionally, SpaiNN offers a calculator module (*calculator.SPaiNNulator*) for interfacing neural network predictions (*via* SchNetPack) with NAMD simulations using SHARC.

The general architecture of SpaiNN is illustrated in Fig. 1. SpaiNN offers several improvements over its predecessor, SchNarc,^33^ most notably by supporting a rotationally equivariant representation of molecules and materials using the paiNN representation in addition to the translationally and rotationally invariant SchNet representation. Furthermore, the code is entirely modular, similar to SchNetPack2.0,^38^ allowing for straightforward addition and modification of modules for predicting excited-state properties. A detailed comparison between SpaiNN and SchNarc is presented in Table S1.†

SpaiNN integrates with SHARC^36^ for ML-driven NAMD simulations. As an interface to SchNetPack 2.0, SpaiNN provides modules for:

• Conversion and generation (from SHARC output files) of databases useable with SpaiNN (*cf.* left column in Fig. 1 and ESI section S1.1 and S1.2†),

• Data pre-processing (*e.g.*, splitting, scaling and transformation; *cf.* ESI section S1.3†),

• Prediction modules for electronic properties in multiple electronic states (*cf.* middle column in Fig. 1), and

• Output modules enabling phase-free training of properties arising from the coupling of two electronic states (*cf.* right column in Fig. 1 and ESI section S1.4†).

Additionally, SpaiNN facilitates communication between SchNetPack 2.0 (ref. 38) and SHARC 3.0 (ref. 37) through the spainn.calculator.SPaiNNulator class. This functionality allows trained ML models to furnish quantum chemical properties (*calculate*) or SHARC predictions (*get_qm*) for molecular structures. A comprehensive description of the data pipeline can be found in the ESI (section S1†) and a code documentation is available as “Read the Docs” page.^39^ In the following, we focus on certain properties that are essential for ML but for a comprehensive overview of the other parts of NAMD simulations with SHARC see ref. 37 and 40.

To account for nonadiabatic transitions, SHARC uses the Tully's fewest switches surface hopping method,^41,42^ which is a mixed quantum-classical method that propagates nuclei classically according to Newton's equation of motion on different excited-state potentials, which are treated by means of electronic structure theory. Nonadiabatic transitions between different PESs are accounted for by so-called hops across different PESs. These are determined stochastically based on the magnitude of the NACs, which are referred to as **C**_*ij*_ in the following and are defined as1
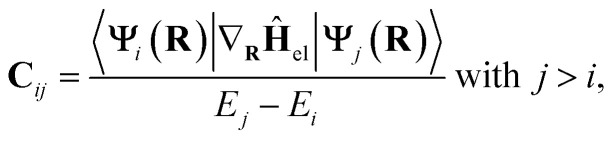
with **Ψ**_*i*_ and **Ψ**_*j*_ representing the electronic wave functions of states *i* and *j* and their respective potential energies *E*_*i*_ and *E*_*j*_, whereas *E*_*j*_ > *E*_*i*_. Fitting these couplings presents significant challenges arising from the intrinsic properties of NACs, characterized by their lack of smoothness and arbitrary sign variations.^43–45^ Specifically, complications arise at conical intersections, critical points in NAMD simulations where the energy levels of electronic states *i* and *j* coincide.^36,46^ However, as electronic states become degenerate, NACs tend toward infinity (*cf.*eqn (1)), giving rise to singularities in their values. To address this issue, SpaiNN incorporates a smoothing process into its pipeline.^45^ This smoothing procedure involves scaling the original NACs by the energy difference between the two states involved in the coupling, denoted as *E*_*j*_ − *E*_*i*_ (*j* > *i*), expressed as

2 **C̃**_*ij*_ = **C**_*ij*_·(*E*_*j*_ − *E*_*i*_).

These modified NACs, referred to as smoothed NACs (**C̃**_*ij*_, ‘*smooth_nacs*’) are pre-computed and stored in the database. This can be achieved by enabling the *smooth_nacs=True* option when using the GenerateDB or ConvertDB classes of *spainn.asetools* to create a SpaiNN database.

Moreover, training of NACs and other properties arising from the interaction of two electronic states is further complicated by the arbitrary phase inherent in the wavefunction, resulting in these properties bearing an arbitrary sign. Numerous strategies have been proposed to confront this challenge, including phase correction of the data prior to fitting,^47,48^ and a phase-free training algorithm enabling the training of raw data.^33^ While the former approach is often applicable only within limited conformational regions of a molecule, the latter is constrained by the escalating training complexity as the number of electronically excited states increases. SpaiNN incorporates two classes of phase-free loss functions, namely *PhaseLossAtomistic* and *PhysPhaseLossAtomistic*, enhancing computational efficiency and simplicity compared to the original approach of SchNarc. The different loss functions implemented are explained in detail in the ESI in section S1.4.†

Besides NACs, energies, forces, and permanent and transition dipole moment vectors can be learned. Forces are treated as derivatives of the potential energy surface with respect to atomic positions and dipole moment vectors are learned using the charge model according to ref. 49 and 50. All properties can be trained simultaneously using a combined loss function:3
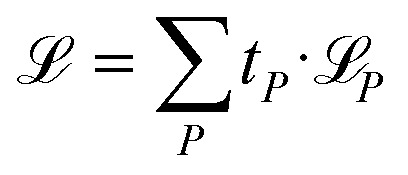
where *t*_*P*_ is a pre-defined parameter to weigh the different properties during training with *P* referring to the set of trained properties, such as energies (*E*), forces (**F**), dipole moments (***μ***), or NACs (**C**). 
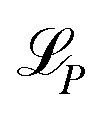
 is the individual loss of each property.

Ultimately, SpaiNN models can be utilized for predicting quantum chemical properties, either independently or within a NAMD simulation using SHARC (*spainn.calculator.SPaiNNulator*). It is important to note that while our models are trained with smoothed NACs (**C̃**_*ij*_), these are converted to classical NACs (**C**_*ij*_) for predictions using the *NacCalculator* class from *spainn.interface.aseinterface*. Specifically, predictions of smoothed NACs and energies for a given geometry are obtained, from which the classical NACs are derived according to eqn (2). Since the *NacCalculator* is employed in our ML-driven surface hopping simulations (within the *spainn.calculator.SPaiNNulator*), classical NACs are used during these dynamic runs. Consequently, the prediction of photochemical properties, such as rates and quantum yields, is expected to be as reliable as surface hopping generally is for predicting these properties.^37,46,51^

## 3 Datasets

Herein, we assess the quality of SpaiNN predictions using two classes of model systems: Firstly, we delve into the light-induced rotation around a double bond across a series of alkenes: ethene (C_2_H_4_), propene (C_3_H_6_), and butene (C_4_H_8_). To achieve this, we created the XAlkeneDB^52^ database at the complete active space self-consistent field (CASSCF) level of theory,^53,54^ specifically averaging the three lowest singlet states targeting two active electrons distributed over two active orbitals, denoted as SA(3)-CASSCF(2,2). A total number of *circa* 6 k (for C_2_H_4_ and C_3_H_6_ each) and 13 k (C_4_H_8_) reference calculations were performed for the respective alkenes, from which the following five quantum chemical features are stored in the XAlkeneDB database: energies, forces, NACs, and dipole moment vectors (comprising both permanent and transition dipole moment vectors). Detailed information regarding the construction of the XAlkeneDB^52^ database is provided in the ESI (*cf.* section S2†).

As second showcase example, we studied the methyleneimmonium cation (CH_2_NH_2_^+^), which is the smallest member of the protonated Schiff bases that has been used as model systems to study the fundamental processes involved in vision.^55,56^ To this end, we relied on the literature-known database of CH_2_NH_2_^+^ comprising 4 k data points at the multi-reference configuration interaction singles and doubles (MR-CISD) level of theory with an active space of four active electrons in three active orbitals, namely MR-CISD(4,3).^47^ This data has served as a good test bed for several developments^33,45,47,57^ as it is characterized by ultrafast internal conversion after excitation to the second excited singlet state.

## 4 Results and discussion

To comprehensively assess the performance of SpaiNN, we investigated two distinct categories of organic model compounds. These molecules share a common characteristic: They show a torsional movement of groups around a C–C bond, specifically a double bond in their ground state, upon absorption of light. This phenomenon arises from the ππ* nature of the reached excited state: When a ππ* state is populated by light, the bond order of the molecules changes to one, converting the double bond to a single σ-bond. Consequently, atoms or groups of atoms can freely rotate around this bond in the electronic excited state, a freedom not available in the ground state. The quintessential example of such a photochemical reaction is the *cis*/*trans*-isomerization.

Understanding these photochemical reactions computationally requires the accurate description of internal conversion between electronic states, particularly accessing NACs at regions of the conical intersections. These intersections are characterized by energetic degeneracy between electronic states, enabling ultrafast, non-radiative relaxation pathways. Consequently, conical intersections serve as critical junctions for light-induced reactions and processes that decide which state is populated. Herein, we evaluate the predictive capability of SpaiNN in determining equivariant, vectorial properties, namely dipole moments (***μ***_*ij*_) and NACs (**C**_*ij*_). The discussion follows a conventional approach in understanding photophysical processes and reactions through computational chemistry. Initially, we focus on static considerations, including the prediction of absorption spectra (Franck–Condon region) and properties of crossing geometries (region of conical intersections). Subsequently, we showcase the application of SpaiNN predictions in conducting photodynamics simulations.

### 4.1 Training and computational efficiency of SpaiNN

To demonstrate the effectiveness of SpaiNN relative to SchNarc in accurately fitting excited-state potentials and properties, we trained SpaiNN models utilizing both SchNet and paiNN representations. Notably, while SpaiNN models utilizing SchNet representation demonstrate comparable performance to SchNarc models, it is important to highlight that the re-implementation of the original SchNarc code within SpaiNN confers increased versatility and computational efficiency.

SpaiNN models are obtained for three alkene molecules, namely ethene (C_2_H_4_), propene (C_3_H_6_), and butene (C_4_H_8_) as compiled in the XAlkeneDB^52^ and methyleneimmonium cation (CH_2_NH_2_^+^) as reported earlier by some of us.^47^ For the three lowest singlet states of C_2_H_4_ and C_3_H_6_, we trained models on energies (*E*_*j*_) and forces (**F**_*j*_, *j* = {0, 1, 2}) as well as the NACs (**C**_01_, **C**_02_ and **C**_12_). Furthermore, we trained models on these nine features and additional dipole moment vectors, namely transition dipoles (***μ***_01_, ***μ***_02_, ***μ***_12_) and permanent dipoles (***μ***_*j*_, *j* = {0, 1, 2}) for C_4_H_8_ and CH_2_NH_2_^+^. Details on hyperparameters of the models are compiled in the ESI (*cf.* section S3.1†).

Table 1 provides an overview of the mean average errors (MAEs) and root mean squared errors (RMSEs) of SpaiNN models utilizing SchNet (white cells) and paiNN (grey cells) representations for fitting these properties for the corresponding four molecules. The errors indicate that the equivariant paiNN representation leads to notable improvements, particularly in predicting vectorial properties compared to SchNet models. Specifically, while the MAEs of energies and forces show only a modest average improvement of about 15% when comparing SchNet and paiNN models, the error in paiNN predicted NACs is on average 1.5 times smaller than that of SchNet models for the four model molecules. This observation is reasonable considering that forces are not learned as vectorial properties, but rather as the first derivative of the energy.^38^ Furthermore, the fact that the equivariant model SpaiNN outperforms SchNarc for all properties is reflected in the MAE error distributions as compiled in Fig. S4, S5, and S10† for each model and property, respectively.

**Table 1:** Mean absolute errors (MAEs) and root mean squared errors (RMSEs) of SpaiNN models for the alkenes ethene, propene, and butene, as well as the methyleneimmonium cation, employing either paiNN (highlighted in gray) or SchNet representations, respectively. The models were trained on energies (*E*_*j*_ in eV), forces (**F**_*j*_ in eV Å^−1^), and non-adiabatic coupling vectors (**C**_*ij*_) involving the three lowest singlet states (S_0_, S_1_, and S_2_). A weight factor of 1.0 was used for energies, forces, and non-adiabatic couplings to ensure a balanced consideration of the target attributes and their trade-offs

	MAE (RMSE)			
	C_2_H_4_	C_3_H_6_	C_4_H_8_	CH_2_NH_2_^+^
*E*_*j*_	0.004 (0.01)	**0.054 (0.08)**	**0.002 (0.01)**	**0.055 (0.11)**
	**0.003 (0.01)**	0.065 (0.11)	0.011 (0.02)	0.091 (0.16)
**F**_*j*_	**0.020 (0.03)**	**0.291 (0.96)**	**0.014 (0.02)**	**0.280 (0.57)**
	0.022 (0.05)	0.409 (0.79)	0.062 (0.09)	0.407 (0.74)
**C**_*ij*_	**0.024 (0.08)**	**0.021 (0.04)**	**0.004 (0.01)**	**0.179 (3.10)**
	0.034 (0.07)	0.031 (0.05)	0.024 (0.06)	0.233 (3.15)
***μ***_*ij*_	—	—	**0.005 (0.02)**	**0.087 (0.19)**
	—	—	0.023 (0.07)	0.125 (0.28)

The computational efficiency of training SpaiNN models and predicting properties using these models is finally evaluated and compared to SchNarc models. Table 2 provides a comparison of training times between SpaiNN and SchNarc models of comparable size (*cf.* hyperparameters in section S3.1†), as well as prediction times for both models on a central processing unit (CPU).

**Table 2:** Training and prediction times for SpaiNN models employing SchNet or paiNN representation. The table reports the times for the whole training and per epoch indicated by the number of epochs in parenthesis as well as the prediction times obtained by averaging the prediction of 1000 structures 100 times. In none of the training procedures the number of maximum epochs (100 k) was reached and the training was stopped earlier when the validation loss was not changing over 300 epochs. Training was performed on a Tesla-P100 GPU (16 GB RAM) using an Intel Xeon Gold 6134 CPU @ 3.20 GHz. Predictions were performed on an Intel(R) Core(TM) i5-12500

Molecules	Training times/s (# epochs)						Prediction times/μs	
	SchNet			paiNN			SchNet	paiNN
C_2_H_4_	53 529	(19 881)	2.7 (1)	14 341	(**6791**)	**2.1** (1)	84.8 ± 1.9	**84.2 ± 2.2**
C_3_H_6_	36 277	(13 821)	2.6 (1)	19 121	(**9061**)	**2.1** (1)	**85.1 ± 1.8**	85.6 ± 2.1
C_4_H_8_	32 755	(7858)	**4.2** (1)	30 709	(**7039**)	4.4 (1)	84.7 ± 2.0	**83.6 ± 2.2**
CH_2_NH_2_^+^	6574	(**2398**)	2.7 (1)	5930	(2770)	**2.1** (1)	85.8 ± 7.6	**85.5 ± 4.5**

The training times displayed in Table 2 demonstrate that paiNN-based SpaiNN models exhibit slightly greater efficiency during training compared to SpaiNN models employing SchNet representation (SchNarc models). This is reflected by training times of 2.1 s per epoch for C_2_H_4_, C_3_H_6_, and CH_2_NH_2_^+^ with paiNN representation, compared to an average of 2.7 s when training SchNet-based SpaiNN models. Conversely, for C_4_H_8_, SchNet-based model training is 0.2 s per epoch faster than paiNN-based model training. However, despite this, paiNN models converge in fewer steps, resulting in overall greater efficiency (8.5 h) compared to training the corresponding SchNet-based model (9.1 h). This efficiency trend could potentially be attributed to the increased data complexity in C_4_H_8_ relative to the other three molecules, given that the database comprises 13 k molecules, while the other databases contain half or less than half of this size (4–6 k data points).

As shown in Table 2, despite the more complex representation employed by SpaiNN, prediction times are nearly comparable when predicting 100 structures. Timings are computed by averaging predictions for 1000 structures over 100 iterations.

### 4.2 Franck–Condon region – prediction of electronic absorption spectra

**Fig. 2:**
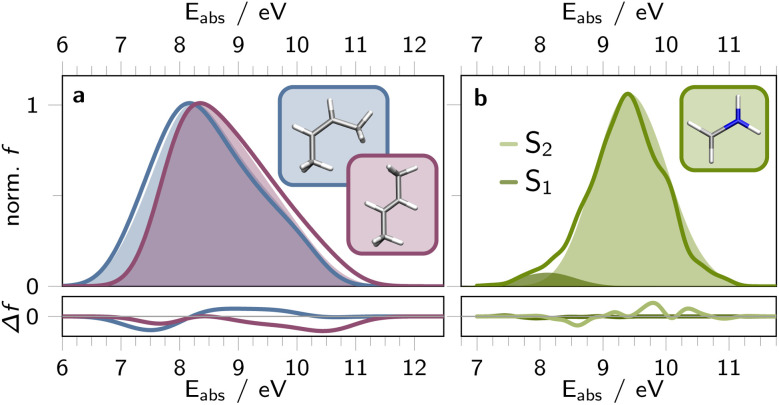
Simulated absorption spectra depicting S_0_ to S_1_ excitation in both *cis*- (blue, a) and *trans*-butene (purple, a), alongside S_0_ to S_1_ (light green, b) or S_2_ excitation (dark green, b) of CH_2_NH_2_^+^. Reference spectra, acquired for 451 geometries of *cis*- and *trans*-butene at SA(3)-CASSCF(2,2)/cc-pVDZ^52^ (a) and for 20 k geometries of CH_2_NH_2_^+^ at MR-CISD(6,4)/aug-cc-pVDZ^49^ (b) level of theory, are represented as solid, unfilled lines. The filled curves display the absorption spectra derived from SpaiNN models (paiNN representation) for the respective structures. The residuals between the SpaiNN- and reference calculated absorption spectra is shown in the bottom panels. Simulated/predicted vertical excitation energies and corresponding oscillator strengths were spectrally broadened using Gaussian functions with a full width at half maximum of 0.1 eV.

In computational investigations of photoinduced reactions and processes, similar to experimental approaches, initial steps often involve characterizing the Franck–Condon region. This entails understanding the energetic and electronic nature of excited states within the framework of the Born–Oppenheimer approximation. Typically, this is achieved by computing vertical transition energies (*E*_abs_) and oscillator strengths (*f*). These parameters quantify the electronic absorption characteristics, whereas the latter is obtained by calculating the energy difference between two electronic states (*i* and *j*) and the 2-norm of the corresponding transition dipole moments (***μ***_*ij*_), as given in eqn (4).4
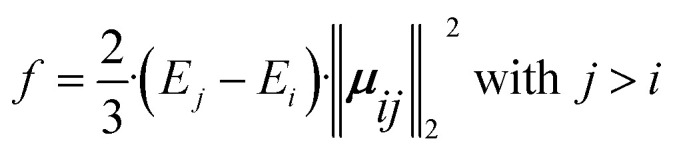
Herein, we selected butene (C_4_H_8_) in both *cis*- and *trans*-configurations, along with the methyleneimmonium cation (CH_2_NH_2_^+^) as our model systems. For predicting absorption spectra we trained a SpaiNN model on energies (*E*_*j*_), forces (**F**_*j*_), NACs (**C**_*ij*_), and dipole moment vectors (***μ***_*ij*_), utilizing both SchNet and paiNN representations. The absorption spectra obtained from quantum chemical reference calculations^49,52^ and SpaiNN predictions are displayed in Fig. 2, S9, and S12.† Notably, including **F**_*j*_ and **C**_*ij*_ in the training is crucial for subsequent simulations of geometries at conical intersections and photodynamics (see section 4.3 and 4.4).

For butene, we conducted reference calculations using SA(3)-CASSCF(2,2)/cc-pVDZ for 451 *cis*- and *trans*-configurations.^52^ These configurations were generated by sampling geometries with varied HC=CH dihedral angles and C=C bond lengths. Specifically, for the *cis*-configuration, we scanned 11 equidistant dihedral angles between 0 to 20° and 41 equidistant alkene bond lengths between 2.0 and 3.0 Bohr. Similarly, for the *trans*-configuration, the same C=C alkene bond lengths were scanned with dihedrals ranging from 160 to 180°. The corresponding absorption spectra are depicted in Fig. 2a as solid, unfilled curves. Additionally, we utilized a SpaiNN model trained on 13 k Wigner sampled structures of butene (*cf.* ESI section S2.1†) for predicting *E*_*j*_ and ***μ***_*ij*_ of these 902 geometries. Fig. 2a illustrates that the ML predicted spectra closely resemble the reference calculations (see filled *vs.* unfilled curves), capturing key spectral features and trends between the *cis*- and *trans*-configurations of butene. Specifically, the maxima of the *cis*- and *trans*-absorption bands align, while the absorption band of the *cis*-configurations appears spectrally broader (by 0.14 eV full-width-half-height) compared to the *trans*-configurations (ref. 0.08 eV difference in full-width-half-height).

Fig. 2b depicts the SpaiNN predicted absorption bands (filled curves) of CH_2_NH_2_^+^ for nπ* (S_0_-to-S_1_, dark green) and ππ* photoexcitation (S_0_-to-S_2_, bright green). The corresponding absorption spectrum obtained for the same 20 000 structures *via* MR-CISD(6,4)/aug-cc-pVDZ is presented as a solid green line in Fig. 2b, as previously reported by some of us.^49^ It is evident that the SpaiNN model of CH_2_NH_2_^+^, accurately predicts absorption bands that closely resemble the reference absorption spectrum.

### 4.3 Static approach – predicting properties across the configurational space

Understanding and predicting excited-state chemistry hinges on thorough knowledge of PESs. However, in polyatomic molecules, PESs are complex high-dimensional functions, posing challenges for characterization. One computational approach – the static ansatz – involves identifying key structures on PESs and establishing reaction paths between them. Typically, this involves sampling various properties across a configurational space to pinpoint important geometries, such as minima and transition states based on potential energies and frequencies, or crossing geometries based on NACs.

This process is computationally intensive, even for molecular systems with few degrees of freedom and electronic states. To streamline this search, ML predictions of properties at *ab initio* quality can be utilized. ML models enable sampling of diverse structures and properties within the boundaries of the configurational space spanned by the training data. In our study, we assess the performance of SpaiNN in predicting properties for the homologous series of alkenes in XAlkeneDB,^52^ namely ethene (C_2_H_4_), propene (C_3_H_6_), and butene (C_4_H_8_), as well as CH_2_NH_2_^+^ with a particular focus on NACs. This emphasis is due to the scarcity of electronic structure methods providing couplings or Hessians and the inherent challenges in fitting NACs in ML approaches due to the phase problem and singularities at conical intersections (*cf.* section 2).

Given the challenge of fitting NACs, we further evaluate the predictive capability of SpaiNN models trained on XAlkeneDB and reference data for CH_2_NH_2_^+^ (ref. 47) by conducting inference on unseen data covering two degrees of freedom. In the first dimension, we varied dihedral angles around the sp^2^ double bond, specifically *δ*(HC=CH) for C_2_H_4_, C_3_H_6_, and C_4_H_8_, or *δ*(HC=NH) for CH_2_NH_2_^+^, within the range of 0 to 180°. In the second dimension, we scanned the length of the respective sp^2^ double bond, ranging between 2.0 and 3.0 Bohr for the alkenes, and between 2.4 and 4.4 Bohr for CH_2_NH_2_^+^.

**Fig. 3:**
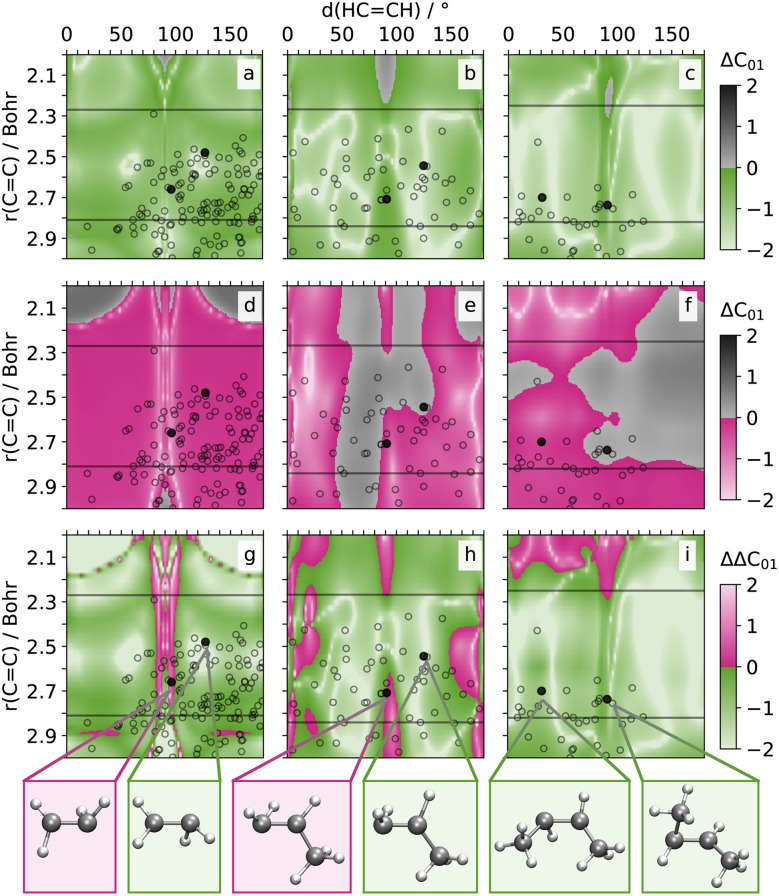
Prediction errors Δ**C**_01_ (a–f): logarithm of the absolute difference between paiNN (a–c) or SchNet-based SpaiNN model predictions and the quantum chemical reference values for nonadiabatic coupling vectors between S_0_ and S_1_ (**C**_01_) for ethylene (a), propylene (b) and butene (c) on a 2D grid along the C=C bond length and (HC=CH) dihedral angles. The prediction errors represent the average values obtained from two paiNN and two SchNet models, which were trained on two different data splits. Colored regions (a–c: green, d–f: pink) indicate prediction errors lower than or equal to 1 atomic unit. Gray regions indicate prediction errors above 1 atomic unit. Divergences in prediction errors ΔΔ**C**_01_ (g–i): difference between the paiNN- and SchNet-based prediction errors, ΔΔ**C**_01_ = Δ**C**_01_ (paiNN) − Δ**C**_01_ (SchNet). Green regions indicate better performance of the paiNN models, while pink regions indicate better performance of the SchNet models. Unfilled symbols (a–i) highlight crossing geometries from SHARC simulations (500 trajectories), where the average CH distance aligns with the 2D grid average CH distance with a certainty of 5%. Selected geometries are shown in the bottom panel, indicated by black filled symbols in the contour plots.

**Fig. 4:**
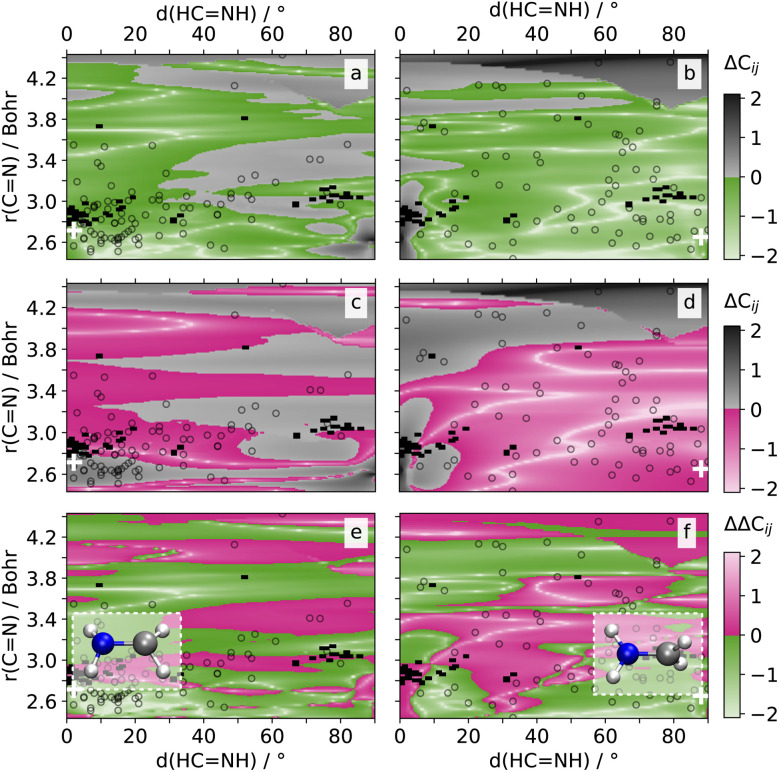
Prediction errors Δ**C**_12_ (a, c) and Δ**C**_01_ (b, d): logarithm of the absolute difference between paiNN (a, b) or SchNet-based SpaiNN model predictions (c, d) and the quantum chemical reference values for nonadiabatic coupling vectors between S_2_ and S_1_ (**C**_01_) and S_1_ and S_0_ (**C**_01_) for the methyleneimmonium cation (CH_2_NH_2_^+^) on a 2D grid along the C=N bond length and (HC=NH) dihedral angles. The prediction errors represent the average values obtained from two paiNN and two SchNet models, which were trained on two different data splits. Colored regions (a, b: green, c, d: pink) indicate prediction errors lower than or equal to 1 atomic unit. Gray regions indicate prediction errors above 1 atomic unit. Divergences in prediction errors ΔΔ**C**_12_ (e) and ΔΔ**C**_01_: difference between the paiNN- and SchNet-based prediction errors, *e.g.*, ΔΔ**C**_12_ = Δ**C**_12_ (paiNN) − Δ**C**_12_ (SchNet). Green regions indicate better performance of the paiNN models, while pink regions indicate better performance of the SchNet models. Unfilled symbols (a–f) highlight crossing geometries from SHARC simulations, where the average CH distance aligns with the 2D grid average CH distance with a certainty of 5%. The white cross-symbols, show the geometry features of the optimized conical intersection geometry in S_2_ (a, c, e) and S_1_ (c, d, f). The respective geometries are shown on top of subsets (e) and (f).

To assess the accuracy of the NAC predictions across the geometries on the two-dimensional grids, we compare the prediction errors obtained for SpaiNN models employing SchNet or paiNN representations. First, we compute the logarithmic, absolute error between each of the models and the quantum chemical reference (Δ**C**_*ij*_, *cf.*Fig. 3a–f and 4a–d). Subsequently, we subtract the SpaiNN(SchNet) from the SpaiNN(paiNN) prediction errors, yielding ΔΔ**C**_*ij*_ = Δ**C**_*ij*_ (paiNN) − Δ**C**_*ij*_ (SchNet) values for every coupling (between states *i* and *j*) at every point on the 2D grid. The respective distribution of these error differences on the 2D grid is shown for ΔΔ**C**_01_ of C_2_H_4_, C_3_H_6_, and C_4_H_8_ in Fig. 3g–i, and for ΔΔ**C**_12_ and ΔΔ**C**_01_ of CH_2_NH_2_^+^ in Fig. 4a and b. The respective error-difference plots for all NACs, potential energies, forces, or dipole moment vectors on the same grid are compiled in Fig. S6–S8 and S11.†

In the difference plots (Fig. 3a–f) the colored areas indicate prediction errors ranging from 1 (dark color) to 0.01 atomic units (bright color) – an error magnitude acceptable for NAMD simulations. *Vice versa*, gray color indicates prediction errors above 1 atomic unit reaching up to 100 atomic units (black). In the corresponding error difference plots, green highlights regions where paiNN-based SpaiNN models yield predictions closer to the reference values compared to those based on SchNet, while pink indicates the opposite. We discuss the respective results for the selected NACs of the alkenes and CH_2_NH_2_^+^ individually in the subsequent paragraphs.

#### 4.3.1 Alkenes

The sampled 2D-grid for the molecules in XAlkeneDB covers the configurational space of interest, *i.e.*, the region around the S_1_/S_0_-conical intersection. Typically, conical intersection-geometries with *δ*(HC=CH) between 80 and 100° and *r*(C=C) ranging from 2.5 to 2.8 Bohr are observed for alkenes. This is supported by the hopping geometries found from SHARC trajectories (#500) for the three alkenes. These are indicated by black symbols in Fig. 3 and refer to geometries, where the average C–H distance aligns with the one of the 2D grid (≈2.05 Bohr) with a certainty of 5%.

The error-difference plots for the alkenes show that paiNN-based SpaiNN models outperform their SchNet-based counterparts in the relevant regions of chemical configuration space for all three alkenes (*cf.* green *vs.* pink color in Fig. 3g–i). While the training data encompasses *δ*(HC=CH) values ranging from 0 to 180°, mirroring the 2D-grid reference points, the C=C double bonds cover distances approximately from 2.25 to 2.80 Bohr in the training set. Consequently, the models interpolate with respect to angles but extrapolate along the distance axis. Nevertheless, it is apparent that SpaiNN models utilizing paiNN representation exhibit superior generalization in regions typically associated with S_1_/S_0_ conical intersections. These intersections, marked by black symbols in Fig. 3, are obtained from SHARC simulations (note: while geometries may not align precisely on the grid, corresponding C=C bond distances and dihedrals are utilized for plotting).

Furthermore, the accuracy of paiNN predictions improves with the increasing size of the alkenes (see white regions in Fig. 3a–c). In contrast, SchNet predictions show a growing number of points with mean errors between 1 and 10 units as the molecule size increases from C_2_H_4_ to C_4_H_8_ (see gray regions in Fig. 3d–f). This trend can be explained by the increasing degrees of freedom in larger molecules, which are more effectively captured by the equivariant models.

In the error difference plots (*cf.*Fig. 3g–i) are certain regions, where the SpaiNN(SchNet) models show a superior performance compared to SpaiNN(paiNN) models. For instance, in case of C_2_H_4_, the SchNet-based predictions of the nonadiabatic couplings are more accurate with respect to the paiNN-based predictions in a region between 2.2 and 2.7 Bohr at dihedral angles between 80 and 100°. Nevertheless, both SpaiNN(SchNet) and SpaiNN(paiNN) exhibit in the regions, which are pink-colored in Fig. 3g–i, mean average errors in the order of 0.01 to 0.1 units, which indicates that both models describe the couplings in this region sufficiently accurately in terms of NAMD simulations.

#### 4.3.2 Methyleneimmonium cation

For CH_2_NH_2_^+^, we consider the NACs of S_2_ and S_1_ (**C**_12_) as well as S_1_ and S_0_ (**C**_01_), since these couplings drive the ultrafast dynamics of the molecule upon photoexcitation into S_2_. To better comprehend the accuracy of the NACs in various regions of the PESs pertinent to NAMD, we plot the differences in the errors of these NACs individually on the 2D-grid along the length of C=N bond and rotation of the H-atom groups around it (*cf.*Fig. 4). These coordinates were found to be pivotal for the conical intersections between the different states (*cf.* structures of optimized S_2_/S_1_ and S_1_/S_0_ conical intersections in Fig. 4a and b).^47^

The first conical intersection between the S_2_ and S_1_ state is characterized by a pyramidalization and a bond distances between 2.65 and 3.40 Bohr mainly, with most hopping geometries located around 2.83 Bohr (see geometries and unfilled symbols in Fig. 4a). Hardly any rotation around the C=N bond is visible in the conical intersection of states S_2_ and S_1_. SpaiNN outperforms SchNarc in NAC accuracy in this region, which is relevant for the S_2_/S_1_ conical intersection, which can be seen by the region of green color Fig. 4a (bottom, left). For completeness, we note that SpaiNN is less accurate at about 90° dihedral angle and large interatomic distances, a region hardly relevant for the dynamics under investigation.^47^

The second conical intersection between the S_1_ and S_0_ state is characterized by a rotation of the C=N bond and a dihedral angle of up to 90°, see also Fig. S4† for a scan along the C–N rotation and corresponding NAC values. Bond distances are mainly at about 2.65 Bohr.^47^ As can be seen in Fig. 4b, the accuracy between SpaiNN and SchNarc models for **C**_01_, *i.e.*, the NAC between the S_0_ and S_1_ states, seem to be balanced. However, the region of interest with small bond distances is mainly characterized by larger errors of SchNet-based SpaiNN models. As the interatomic distance increases, SchNet-based SpaiNN models exhibit greater accuracy compared to paiNN-based counterparts. However, the relevance of NACs diminishes for the investigated dynamics. For completion, the error difference in NACs between the S_0_ and S_2_ state as well as the energies and dipoles is plotted in Fig. S11.† As evident from this figure, the majority of regions are shaded green, indicating that SpaiNN models utilizing paiNN representation consistently outperform their counterparts with SchNet representation across all examined NACs, energies, and dipoles and nearly all areas of the investigated 2D PES.

### 4.4 Dynamic approach – predicting ultrafast internal conversion

Given the limitations of static photochemistry approaches in potentially overlooking crucial geometries and pathways as well as relaxation mechanisms, time scales and quantum yields, we integrated SpaiNN with SHARC 3.0.^37^ The latter forms the methodological foundation for dynamic approaches employing surface hopping NAMD. To showcase the performance of SpaiNN-assisted SHARC simulations, we employed the SpaiNN models of C_4_H_8_ and CH_2_NH_2_^+^ to conduct photodynamics simulations of the respective molecules. For the latter molecule, this has been used to assess the accuracy of NAMD methods earlier as the accurate description of its photodynamics that are governed by ultrafast internal conversions between different electronically excited states require accurate NACs.^45,47,57^

To execute ML-photodynamics we employed the SchNet- and paiNN-based SpaiNN models of C_4_H_8_ and CH_2_NH_2_^+^ trained on energies (*E*_*j*_), forces (**F**_*j*_), NACs (**C**_*ij*_), and dipole moment vectors (***μ***_*ij*_) as discussed in section 4.1 (*cf.*Table 1). Note that ***μ***_*ij*_ were included and trained for completeness, although they remain inactive in the photodynamics simulations under discussion. Nevertheless, their potential application lies in the prediction of electronic absorption spectra,^49^ thereby facilitating the selection of initial conditions within the conventional framework of NAMD simulations as discussed above (*cf.* section 4.2).

**Fig. 5:**
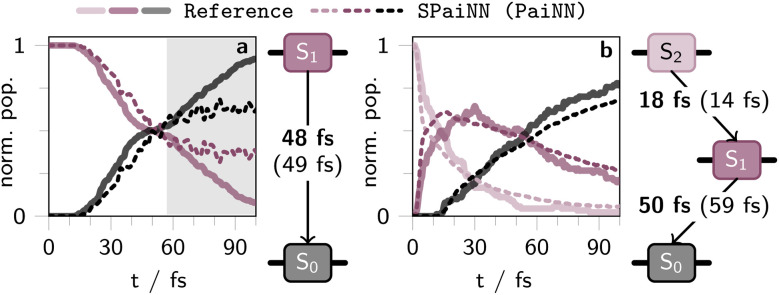
Temporal evolution of population in the two lowest singlet excited states upon photoexcitation into S_1_ of *cis*-butene (a) and the three lowest singlet excited states of CH_2_NH_2_^+^ upon excitation into S_2_ (b) as derived from SHARC simulations spanning 100 fs (with a time-step of 0.5 fs). The solid lines represent outcomes from 100 SHARC trajectories on SA(3)-CASSCF(2,2)/cc-pVDZ (a) or MR-CISD/aug-cc-pVDZ level of theory, respectively.^47,52^ Dashed lines reflect results from 600 (a) or 3846 SHARC trajectories (b), incorporating non-adiabatic couplings, energies, and forces predicted by a SpaiNN model leveraging the equivariant paiNN representation. The background color is white in regions where SpaiNN models yield reasonable results, as determined by the total energy of the molecules at each time-step, while other regions are shaded gray. Kinetic rate constants for a sequential kinetics scheme were derived using KiMoPack^58^ and are presented alongside the population plots. Bold numbers denote the excited-state lifetimes as obtained from reference calculations, the ML lifetimes are given in parenthesis.

The results of the paiNN-based SpaiNN predictions of the photodynamics of C_4_H_8_ and CH_2_NH_2_^+^ are summarized in form of kinetic plots showing the population of the two (C_4_H_8_) or three lowest singlet excited states (CH_2_NH_2_^+^) in Fig. 5a and b, respectively. The corresponding results obtained with SchNet-based SpaiNN models are shown in Fig. S14.†

For both molecules, it is evident from the population curves in Fig. 5 that the SpaiNN-driven SHARC simulations are able to reproduce the ultrafast transitions between the different electronic states as obtained from SHARC reference calculations (*cf.* computational details in section S2.4). This is reflected in the match between the dashed (SpaiNN/SHARC simulations) and the solid lines (electronic structure theory/SHARC simulations) in the white shaded regions in Fig. 5.

For CH_2_NH_2_^+^ (Fig. 5b), the transition from the first excited state (S_1_) to the ground state (S_0_) is marginally better captured when comparing paiNN- and SchNet-based SpaiNN models evaluated against quantum chemistry reference simulations.^47^ This is evidenced by the characteristic lifetime of S_1_ obtained from a sequential kinetic model, which is 30 fs and 50 fs for SchNet- and paiNN-based SpaiNN predictions, respectively, whereas the reference value is 59 fs. Nevertheless, employing both SchNet and paiNN-based SpaiNN models in SHARC simulations yields reasonable photodynamics results, aligning with previous analyses and findings from related studies.^45^

In contrast, photodynamics simulations for C_4_H_8_ employing paiNN-based SpaiNN models outperform those using SchNet-based counterparts. However, both models exhibit reliability limitations, constrained to a simulation time of approximately 60 fs, as indicated by the gray region in Fig. 5 and S14,† determined by the total energy of the molecules at each time-step of the dynamics simulation. This is due to the fact that the C_4_H_8_ database was not optimized through active or online learning, hence the simulations fail for both SchNet- and paiNN-based SpaiNN models after reaching a certain simulation time, corresponding to long-tail events not well represented in the training data. Only the paiNN-based SpaiNN model accurately reproduces the population kinetics of butene with respect to the reference, yielding a lifetime of the initially excited S_1_ state of 49 fs, consistent with reference calculations (48 fs), while SchNet-based models yield a lifetime of about 15 fs (Fig. S14a†). This underscores the influence of the more accurate excited-state properties of paiNN compared to SchNet-based SpaiNN models.

## 5 Conclusions

This work introduces SpaiNN, an open-source Python software for performing surface hopping molecular dynamics simulations utilizing machine learning predicted energies, forces, nonadiabatic couplings or dipole moment vectors. By the integration of the nonadiabatic molecular dynamics program SHARC 3.0 with the neural network potentials provided by SchNetPack 2.0, SpaiNN provides a flexible and modular tool for training and predicting excited state properties involving multiple electronic (singlet) states using rotationally-invariant (SchNet) or -equivariant (paiNN) representation of molecules. Moreover, its modular architecture allows for straightforward adaption of modules and the development of new ones. The method is accessible on GitHub^59^ and is accompanied by a comprehensive documentation^39^ including tutorials, which facilitate its integration into research. SpaiNN incorporates three core modules to (i) generate databases or convert existing databases into the SpaiNN database format, and to interface (ii) SchNetPack and (iii) SHARC.

The performance and capability of SpaiNN is demonstrated using simulations of the methyleneimmonium cation and a series of alkenes. The results show improved accuracy and comparable computational efficiency to previous methods (SchNarc), particularly in fitting non-adiabatic couplings, energies, and forces of multiple electronic excited singlet states. Overall, we show that the usage of the equivariant representation, improves the data efficiency in training and inference as well as the generalization of the respective models. We believe that SpaiNN is promising for the study of various photophysical and photochemical phenomena and the comprehensive documentation and tutorials available with this method facilitate the development of new interatomic potentials for molecules in their excited states.

## Data availability

The reference and training data of the alkenes studied in this article, including the energies, forces, and permanent dipole moments of S_0_, S_1_, and S_2_ and the transition dipole moments and non-adiabatic couplings between these states on SA(3)-CASSCF(2,2)/cc-pVDZ level of theory are available at Zenodo at https://doi.org/10.5281/zenodo.10736050. The data of the methylene immonium cation was previously published by some of us and is available on Github at https://github.com/schnarc/SchNarc and RSC at https://doi.org/10.1039/C9SC01742A. SPaiNN, including the source code, documentation and tutorials is available at Github at https://github.com/CompPhotoChem/SpaiNN.

## Author contributions

S. M. and C. M. conducted the main programming work, co-wrote the code documentation and co-developed tutorials. C. M. performed the quantum chemical calculations to create the XAlkeneDB database. J. W., C. M. and P. M. conceived the study. All authors contributed to the writing of the manuscript, provided feedback, and edited it.

## Conflicts of interest

There are no conflicts to declare.

## Supplementary Material

SC-OLF-D4SC04164J-s001
