# Supplementary material for: SpaiNN: equivariant message passing for excited-state nonadiabatic molecular dynamics
Source: Chem Sci. 2024 Sep 2;15(38):15880–90. doi: 10.1039/d4sc04164j (PMC11391904; doi:10.1039/d4sc04164j)
Supplement: SC-015-D4SC04164J-s001 [file SC-015-D4SC04164J-s001.pdf]

Supplementary Information (SI) for Chemical Science.
This journal is © The Royal Society of Chemistry 2024

Supporting Information: SPAINN: Equivariant Message Passing for Excited-State Nonadiabatic Molecular Dynamics

Sascha Mausenberger,[a,f‡] Carolin Müller,[*b,c‡] Alexandre Tkatchenko,[b] Philipp Marquetand,[a] Leticia González[a] and Julia Westermayr[*d,e]

Contents

S1 The SpaiNN Method 2
S1.1 Data Pipeline . . . 2
S1.2 Creating a data base . . . 3
S1.3 Data Pre- and Postprocessing . . . 3
S1.4 Phase-Free Training . . . 4

S2 Databases 6
S2.1 XAlkeneDB[1] . . . 6
S2.2 Methylenimmonium cation ($CH_2NH_2^+$) . . . 9

S3 SpaiNN Models 10
S3.1 Parameters . . . 10
S3.2 Performance: Training on XAlkeneDB . . . 10
S3.3 Performance: Training on $CH_2NH_2^+$ database . . . 15

[a] *University of Vienna, Institute of Theoretical Chemistry, Faculty of Chemistry, Währinger Str. 17, 1090 Vienna, Austria*
[b] *University of Luxembourg, Department of Physics and Materials Science, 162 A, Avenue de la Faïencerie, L-1511 Luxembourg, Luxembourg*
[c] *Friedrich-Alexander-Universität Erlangen-Nürnberg, Department Chemistry and Pharmacy, Computer-Chemistry-Center, Nägelsbachstraße 25, 91052 Erlangen, Germany*
[d] *Leipzig University, Wilhelm Ostwald Institute for Physical and Theoretical Chemistry, Linnéstraße 2, 04103 Leipzig, Germany*
[e] *Center for Scalable Data Analytics and Artificial Intelligence (ScaDS.AI), Dresden/Leipzig, Germany*
[f] Vienna Doctoral School in Chemistry (DosChem), University of Vienna, Währinger Straße 42, 1090 Vienna, Austria

S1 The SpaiNN Method

The SPAINN method enables computationally efficient machine learning (ML)-driven nonadiabatic molecular dynamics (NAMD) simulations with SHARC[2,3] using equivariant representations for molecules and materials in a message-passing deep neural network.[4] The SPAINN method is available on github[5] and includes a full documentation and tutorial[6] (*cf.* Figure S1).

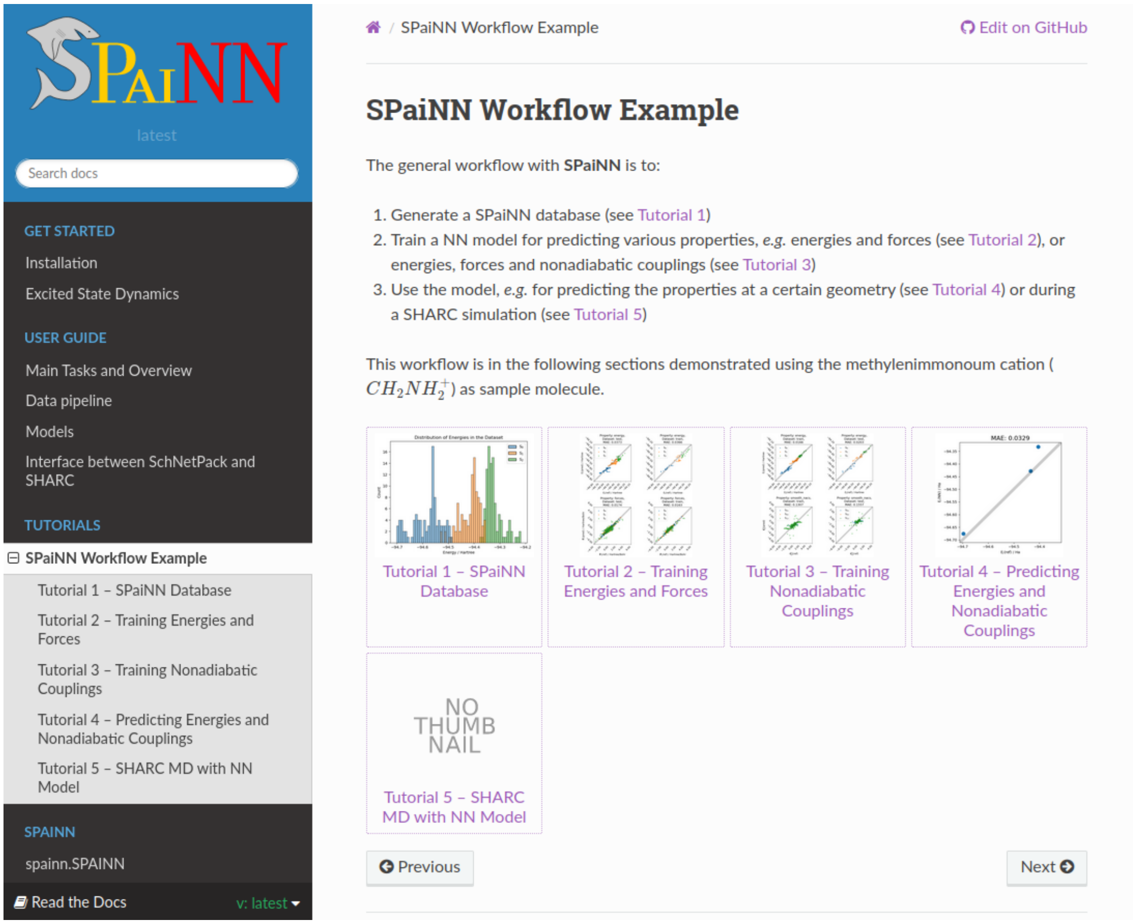

Fig. S1 ReadTheDocs: Illustration of the documentation and tutorials available for SPAINN.[5]

S1.1 Data Pipeline

As shown in Figure 1 in the main text, SPAINN is built in a modular way. The first pipeline is the data pipeline that consists of two primary components: *i)* the creation of a SPAINN data base, and *ii)* the PyTorch data base interface and data loader. The latter component is essentially an extension of the AtomsDataModule from SchNetPack 2.0.[7] It incorporates a multi-state statistics option, allowing the calculation of mean values and standard deviations for multiple electronic states. We will first delve into the details of the data base aspects, followed by a brief explanation of the datamodule and how we expanded the pre- and postprocessing functions to accommodate multiple electronic states.

S1.2 Creating a data base

A SpaiNN data base can be created using GenerateDB or ConvertDB: The purpose of GenerateDB is to generate a SpaiNN-type data base in the Atomic Simulation Environment (ASE)[8] format. This data base is generated based on the output of SHARC,[3] which includes relevant information such as Hamiltonian, transition dipole moment matrices, and nonadiabatic coupling vectors (NACs) obtained from a QM.out file, if created with SHARC.[3] The respective database is essentially an ASE database[8], but there are certain requirements for metadata information and the dimensions in which the quantum chemical properties are stored to enable a smooth interface with SchNetPack 2.0.[7]

Scalar and vectorial quantum chemical properties are stored in a shape of $(N_a \times N_k)$ or $(N_a \times N_k \times 3)$, respectively, where N_k refers to the number electronic states (N_S) or couplings between them, $N_C = 0.5 \cdot N_S \cdot (N_S - 1)$. For atomistic properties (*e.g.*, forces and non-adiabatic couplings), N_a refers to the number of atoms, while for collective properties (*e.g.*, energy and transition dipole moments) $N_a = 1$.

Moreover, during the generation of a data base, the total number of electronic states (N_S) and the respective number of singlet, doublet, and triplet states as well as a dictionary of units for all properties and the atomic coordinates are added in form of metadata to the SpaiNN data base. Including the units of properties ensures easy conversion of units without requiring internal default units. The default values are summarized in the following Python code snippet:

```
distance_unit = "Bohr"
property_unit_dict = {"energy": "Hartree",
                      "forces": "Hartree/Bohr",
                      "nacs": "1",     # non-adiabatic couplings
                      "dipoles": "1", # dipole momenta
                      "socs": "1"}     # spin-orbit couplings
```

A SpaiNN data base db_spainn.db from QM.out files located in a folder data can be created by the following code using Python API:

```
import spainn
from spainn.asetools import GenerateDB
genDB = GenerateDB()
genDB.generate(path='data',
               dbname=os.path.join('data', 'db_spainn.db'),
               smooth_nacs=True)
```

or using the Command Line API:

```
spainn-db generate ./data/ ./data/db_spainn.db
```

On the other hand, ConvertDB is responsible for converting an existing ASE data base into a SpaiNN-type data base (*e.g.*, reshape properties and add metadata). For instance, when converting the NACs of an existing SchNarc data base into SpaiNN format, the property dimensions are shifted from $(N_S \times N_a \times 3)$ to $(N_a \times N_S \times 3)$.

S1.3 Data Pre- and Postprocessing

SchNetPack provides PyTorch modules without trainable parameters that are used to preprocess inputs before batching and postprocess model results, *i.e.*, acts on batches. Naturally, every SchNetPack model requires the construction of a neighbor list in a postprocessing step.

Before building the model, we remove offsets from the energy for good initial conditions. We will get this from the training data base. Above, this is done automatically by the RemoveOffsets transform.

An additional option with respect to SchNarc is that during the generation or conversion of a data base, smooth NACs can be added to the data base. As previously described, the direct learning of NACs is a challenging ML task, since the overall shape of the couplings (including singularities and cusps) is difficult to fit. The way of smoothing these sharp features is by multiplying the coupling values between two states with the respective energy difference of these two states, which reads as

$$\tilde{C}_{ij} = C_{ij} \cdot (E_j - E_i) \;\; \text{with } j > i \; . \tag{1}$$

S1.4 Phase-Free Training

Predicting excited state properties presents a challenge due to the need to consider multiple electronic states and their associated couplings ($\mathbf{Q}_{ij}$), such as NACs ($\mathbf{C}_{ij}$) or transition dipole moments (μ_{ij}). The prediction of these properties, that arise from two electronic wave functions has proven to be difficult when utilizing ML techniques.[9,10] This is because the phase of each coupling property depends on the sign of the wave functions of the electronic states i and j, that are coupled by this property, which however is not uniquely defined in quantum chemical calculations. Consequently, there can be random phase jumps in the coupling values along a reaction path. Thus, the couplings cannot be learned directly.

To address this issue, some of us have lately established an approach to eliminate the influence of the arbitrary phase during the learning process of the ML model.[9] The core idea of this phase-free training lies in the definition of a loss function ($\mathscr{L}_P$), which internally performs a phase correction. This is realized by computing the loss $2^{N_\mathrm{S}-1}$-times, *i.e.*, for all possible phase combinations of all coupled electronic states (N_S) of the prediction and find the one that has the lowest error compared to the target.

Briefly, for a given number of states (N_S), we consider all possible couplings between them, namely $N_C = 0.5 \cdot N_S \cdot (N_S - 1)$. Each coupling property $\mathbf{Q}_{ij}$ can have either a positive or negative sign, depending on the sign of the wave function of the two electronic states, i and j, that are coupled by this property. To capture the full range of sign combinations, we generate in the `PhaseLossMAE` and `PhaseLossMSE` loss function phase vector permutations. As a reference, we assign a fixed positive sign, *i.e.*, a value of $+1$ to a selected coupling. Consequently, the total number of permutations N_P is 2^{N_S-1}. In this way an $(N_C \times N_P)$-matrix ($\mathbf{p}$) including all phase vector permutations is constructed. Ultimately, the model predictions $\mathbf{Q}^{\mathrm{NN}}$ element-wise multiplied with $\mathbf{p}$ enter the loss function, and from the resulting $(N_c \times 2^{N_S-1})$-matrix, the column giving the lowest loss value is handed to the ML model and further optimized. The respective phase-free loss function – implemented as `spainn.loss.PhysPhaseLossAtomistic` – reads as

$$\mathscr{L}_p = \frac{1}{3N} \min_i \left(\sum_j^{N_\mathrm{C}} \sum_\omega \left| \mathbf{Q}^{\mathrm{ref}} \mathbb{1}_{N_\mathrm{p}}^\top - \mathbf{Q}^{\mathrm{NN}} \mathbb{1}_{N_\mathrm{p}}^\top \odot \mathbf{P} \right|_{aij}^m \right) \tag{2}$$

$$\text{with } a \in \omega, \; m \in \{1,2\} \; .$$

$$(\omega, N) = \begin{cases} ((l),\, N_\mathrm{C}) & \text{(bulk)} \\ ((k,l),\, N_\mathrm{C} \cdot N_\mathrm{A}) & \text{(atomistic)} \end{cases}$$

Here, the summation is performed using the indices j, k, and l. The values of j range from 1 to N_C, k ranges from 1 to N_at, and l ranges from 1 to 3 (xyz-direction). To avoid redundant calculations, we developed a new approach for phase-free training based on the established loss function of SchNarc (equation 2). Instead of trying to phase correct for all coupling properties at once using $2^{N_\mathrm{S}-1}$ combinations, we treat each coupling individually. This is realized by multiplying each coupling with $+1$ or -1 before computing the loss with respect to the target value. Ultimately, the sum of the lowest loss of each coupling enters the overall loss value. The respective updated loss function – implemented as `spainn.loss.PhaseLossAtomisticMSE`

($m = 2$) and `spainn.loss.PhaseLossAtomisticMAE` ($m = 1$) – reads as

$$\mathscr{L}_p = \frac{1}{3N} \sum_j^{N_\mathrm{C}} \min_i \left(\sum_\omega \left| \mathbf{Q}_j^{\mathrm{ref}} \mathbb{1}_2^\top - \mathbf{Q}_j^{\mathrm{NN}} \mathbb{1}_2^\top \odot \begin{pmatrix} 1 \\ -1 \end{pmatrix}^\top \right|_{ai}^m \right) \tag{3}$$

The new implementation provides similar results in terms of accuracy and computational efficiency for training excited-state properties of the methylenimmonium cation and butene and if subject to extensive testing in the future.

When training multiple properties, the total loss is computed as weighted sum of all losses according to

$$\mathscr{L}_{\mathrm{tot}} = \sum_i^{N_\mathrm{P}} t_i \cdot \mathscr{L}(\mathrm{P}_i) \ , \tag{4}$$

where t_i and $\mathscr{L}(\mathrm{P}_i)$ define the trade-offs and loss value of each property involved in the training (N_P), respectively.

Table S1 Comparison between SchNarc[11] (which combines SchNet with SHARC) and SPaiNN (which integrates SchNet and PaiNN with SHARC).

	SPaiNN	SchNarc
Code	• modular<br>• module for training<br>• module for data base generation<br>• improved efficiency for loss function<br>• configuration files for training	• non-modular<br>• separate script for training<br>• separate script for data base generation
Documentation	• tutorial Juypter Notebooks<br>• comprehensive Read-the-Docs	• tutorial Juypter Notebooks
Representation	• SchNet<br>• PaiNN	• SchNet
Energy prediction	• as scalar properties	• as scalar properties
Force prediction	• as derivatives of energies	• as derivatives of energies
Dipole moment vector prediction	• via charge model[11]<br>• direct	• via charge model[11]
Prediction of NACs	• via smoothed NACs<br>• as derivatives of virtual properties<br>• direct	• via smoothed NACs<br>• as derivatives of virtual properties
Phasecorrection	• via data preprocessing<br>• inside loss function using<br>∘ the phase vector (exponential scaling with N_{states})<br>∘ multiplication with $+1$ and -1 (linear scaling N_{states})	• via data preprocessing<br>• inside loss function using<br>∘ the phase vector (exponential scaling with N_{states})

S2 Databases

S2.1 XAlkeneDB [1]

The pipeline for generation of the XAlkeneDB can be divided into three key steps: **i)** The generation of an initial set of molecules with HC=CH dihedral angles ranging from 0 to 180°, **ii)** the sampling of displaced geometries along that path, and **iii)** the computation of the electronic properties of these structures. The details of the workflow are outlined in the following.

i) Initial molecular geometries

Firstly, the equilibrium geometries of the three molecules in the XAlkeneDB dataset, namely ethene (C_2H_4), propene (C_3H_6) and butene (C_4H_8) were determined. Notably, for butene, the geometry of the *cis*-isomer, which is energetically less favorable than the global minimum structure (*trans*-butene), was also optimized. The equilibrium geometries were obtained using density functional theory (DFT) by means of the hybrid functional `PBE0` [12,13] in conjunction with the `def2-TZVP` basis [14] and Grimme-Dispersion with Becke-Johnson damping [15] as implemented in the `orca 5.0.3` [16] software package. A vibrational analysis was conducted to confirm that the obtained geometries correspond to minima on the potential energy surface.

To expand the configurational space beyond the global/local minima on the potential energy surface, we initially sampled structures with HC=CH dihedral angles (θ) ranging from 0 to 180° (*cf.* cross-marks in Figure S2i). For butene, we interpolated geometries between the optimized *cis*- ($\theta = 180°$) and *trans*-isomer ($\theta = 0°$), producing 11 additional structures with HC=CH dihedral angles of 18, 36, 54, 72, 81, 90, 99, 108, 126, 144, and 162° (*cf.* Figure S2 (i)). Notably, in the vicinity of the typical S_1/S_0 conical intersection for photoinduced isomerization, particularly around θ of 90°, we sampled the path in smaller increments of 9° instead of 18° steps. Since ethene and propene lack *cis* and *trans*-isomers and due to symmetry reasons, we generated five additional geometries for these molecules with HC=CH dihedral angles of 18, 36, 54, 72, and 90°, in addition to their equilibrium structures ($\theta = 0°$). For geometry interpolation, the Nudged Elastic Band (NEB) [17] approach as implemented in `orca` was employed using `PBE0/def2-TZVP` [12–14] level of theory.

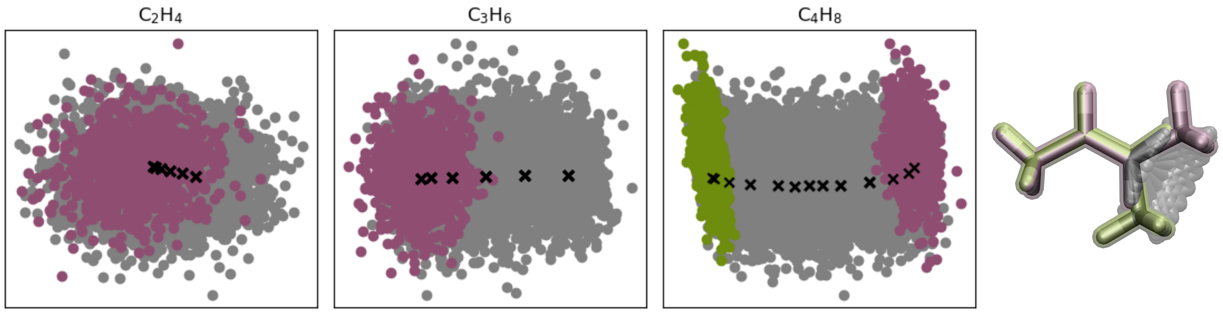

(i) PCA plots. **(ii)** Path (crosses) for C_4H_8.

Fig. S2 Two-dimensional principal component analysis plots (i) for a comparison of the configuration space distributions spanned for C_2H_4 (left), C_3H_6 (middle), and C_4H_8 (right) in the XAlkeneDB dataset. The cross-marks indicate the scanned path around the HC=CH dihedral angle (in steps of 18° between 0 and 180°) to generate an initial set of 6 (C_2H_4 and C_3H_6) or 13 geometries (C_4H_8). Based on these geometries, a set of 1000 structures was sampled by means of Wigner sampling (grey dots). The 1 k ensemble of the geometries sampled for the minimum geometry of the initial path is indicated in purple. In case of butene, the ensemble sampled around the *trans*- and *cis*-isomer equilibrium geometry are indicated in purple and green, respectively (see structures in panel ii).

ii) Molecular geometries in the database

To broaden the configurational space beyond alkene bond rotations, we employed Wigner sampling to encompass vibrational degrees of freedom. Initially, we sampled geometries (6 for ethene/propene and 13 for

butene), covering rotations around the alkene double bond. These geometries and their associated harmonic frequencies (*cf.* previous section) serve as the foundation for generating a set of non-equilibrium structures sampled from a Wigner probability distribution function.[18,19] In brief, each of the $3N-6$ normal mode coordinates and momenta ($N\ldots$ number of atoms) is randomly sampled according to a probability distribution function. The respective sampled normal-mode coordinates and momenta are then converted into Cartesian coordinates and velocities. The Wigner sampling calculations were performed with the initial conditions program integrated into the SHARC 3.0 package.[20,21]

Using the normal mode coordinates of the PBE0 equilibrium and non-equilibrium structures as input for the Wigner distribution, we generated a total of 6,000 and 13,000 geometries for ethene/propene and butene, respectively (*cf.* Figure S2). These geometries were equally distributed into the different configurations on the initial path scanning the alkene bond rotation. For example, for butene, 1000 geometries were created for the *cis* and *trans*-isomer and the nine interpolated geometries that connect these two isomers.

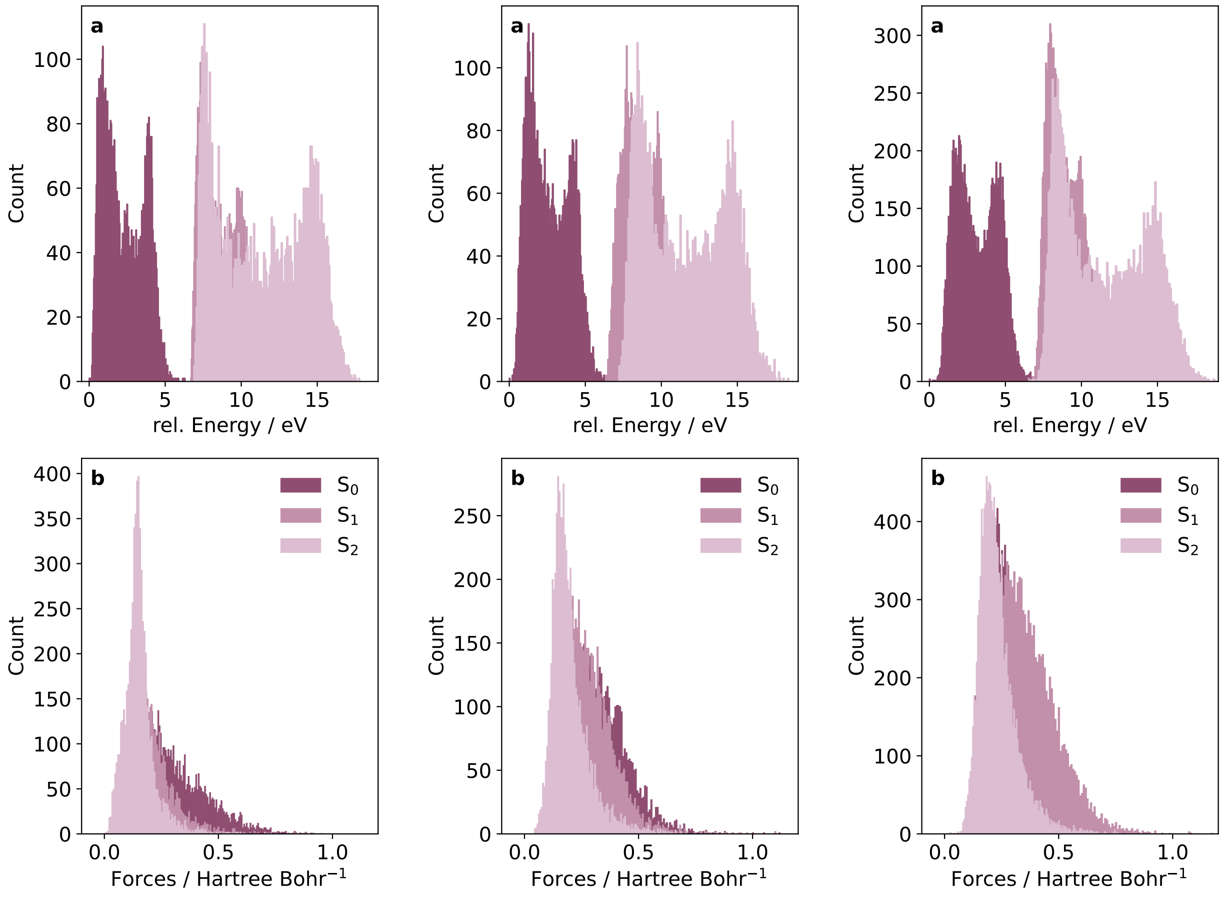

(i) Ethene (C_2H_4) **(ii)** Propene (C_3H_6) **(iii)** Butene (C_4H_8)

Fig. S3 Statistical distribution of the potential energies (top row, a) and Frobenius norm of forces matrices (bottom row, b) of three lowest lying singlet-states (S_0, S_1, and S_2) of C_2H_4 (left), C_3H_6 (middle), and C_4H_8 (right), *i.e.*, the molecules of the XALKENENDB dataset. Energy values are relative to the minimum in the ground-state (S_0) of each dataset. The histograms depict the overall count of data points within each subset of molecules from the XALKENENDB dataset, utilizing 150 bins across the entire range.

iii) Single-point calculations

Lastly, the ground- and excited-state properties for all 25,000 geometries were obtained using the complete active space self-consistent field (CASSCF) approach[22,23] in conjunction with the double-ζ cc-pVDZ[24] basis set as implemented in OpenMolcas[25]. For this purpose, an active space of two active electrons in two orbitals, including the π- and π^*-orbitals of the C=C double bond was chosen. The electronic properties of the individual three lowest lying singlet states (S_0, S_1, and S_2) and their couplings were obtained using state-averaging of these three singlet states — *i.e.* SA(3)-CASSCF(2,2).

Ultimately, the potential energies (`'energy'`: E_0, E_1, E_2), atomic forces (`'forces'`: $\mathbf{F}_0$, $\mathbf{F}_1$, $\mathbf{F}_2$) and permanent dipoles (`'dipoles'` (diagonal): μ_0, μ_1, μ_2) in the three singlet-states S_0, S_1, and S_2 as well as the transition dipole moments (`'dipoles'` (off-diagonal): μ_{10}, μ_{20}, and μ_{21}) and NACs (`'nacs'`: $\mathbf{C}_{10}$, $\mathbf{C}_{20}$, and $\mathbf{C}_{21}$) of every structure (cartesian coordinates) are stored in the XAlkeneDB dataset (*cf.* Table S2). Exemplarily, the statistical distribution of energies and forces in the XAlkeneDB database are illustrated in Figure S3.

Table S2 Summary of electronic properties stored in the XAlkeneDB, detailing the property type, corresponding dictionary key, and property shape within the database. N_{atoms} denotes the total number of atoms, N_{states} indicates the number of states (here $N_{states}=3$: S_0, S_1, and S_2), and $N_{couplings}$ represents the number of couplings (here $N_{couplings}=3$: S_2/S_1, S_1/S_0, and S_2/S_0).

Property	Key	Shape	Comment
potential energy (E_j)	`'energy'`	$(1, N_{states})$	
atomic forces ($\mathbf{F}_j$)	`'forces'`	$(N_{atoms}, N_{states}, 3)$	
dipole moments	`'dipoles'`	$(N_{states}+N_{couplings}, 3)$	
permanent dipoles (μ_j)	`'dipoles'`	$(N_{states}, 3)$	data['dipoles'][$: N_{states}+1$]
transition dipoles (μ_{ji})	`'dipoles'`	$(N_{couplings}, 3)$	data['dipoles'][$N_{states}+1:$]
non-adiabatic coupling vectors ($\mathbf{C}_{ji}$)	`'nacs'`	$(N_{atoms}, N_{couplings}, 3)$	
smoothed non-adiabatic coupling vectors ($\tilde{\mathbf{C}}_{ji}$)	`'smooth_nacs'`	$(N_{atoms}, N_{couplings}, 3)$	$\tilde{\mathbf{C}}_{ji} = \mathbf{C}_{ji} \cdot (E_j - E_i)$ with $j > i$

iv) Grid-extension of XAlkeneDB

To evaluate the performance of ML models trained on XAlkeneDB with out-of-training samples, we conducted quantum chemical reference calculations on a 2D-potential geometrical space comprising 3731 points (41×91 points). This grid was constructed based on the most stable equilibrium geometry of ethene, propene, and butene, respectively. We scanned the distance of the alkene C=C bond between 2.000 and 3.000 Bohr in 41 steps of 0.025 Bohr, and varied the HC=CH dihedral angle from 0 to 180° in 2° increments (91 steps). For each geometry, electronic properties were obtained at the SA(3)-CASSCF(2,2)/cc-pVDZ level of theory for the three lowest-lying singlet states, as detailed above (**iii**). The corresponding data is available in the XAlkeneDB, identified by the string `'grid'` within the subset name.

S2.2 Methylenimmonium cation ($CH_2NH_2^+$)

4,000 reference data points of the methylenimmonium cation ($CH_2NH_2^+$) were taken from the database as described in reference 10. In brief, the electronic properties of these points were obtained by means of multi-reference configuration interaction method including single and double excitations approach – employing an active space of 6 electrons in 4 orbitals – in conjunction with the aug-cc-pVDZ[24,26] basis set — *i.e.* MRCISD(6,4)/aug-cc-pVDZ.

i) Surface hopping molecular dynamics of the methylenimmonium cation

For surface hopping molecular dynamics simulations quantum chemical populations are obtained from 100 trajectories of ref. 10. These populations are obtained by means of MRCISD(6,4)/aug-cc-pVDZ for the three lowest-lying singlet states. For ML-driven molecular dynamics, 3846 trajectories with initial conditions obtained from the same reference were simulated. In all cases, a time step of 0.5 fs was employed. Hopping probabilities were computed based on the NAC strengths between the potentials. Decoherence correction based on the SHARC 2.0 package[2,3] was applied in all cases.

ii) 2D Potential energy surface

To evaluate the performance of ML models trained on the data of $CH_2NH_2^+$, taken from reference 10, with out-of-training samples, we conducted quantum chemical reference calculations on a 2D-potential energy surface comprising 9191 points (101×91 points). This grid was constructed based on the geometry of $CH_2NH_2^+$ in the reference database with the lowest energy in the ground state. We scanned the distance of the C=N bond between 2.432 and 4.432 Bohr in steps of 0.020 Bohr (101 steps), and varied the HC=NH dihedral angle from 0 to 90° in 1° increments (91 steps). For each geometry, electronic properties were obtained at the SA(3)-MRCI(6,4)/aug-cc-pVDZ level of theory for the three lowest-lying singlet states, as detailed above (**i**).

S3 SpaiNN Models

S3.1 Parameters

To train the SpaiNN models, we initially obtained initial guesses from default values[9,11] and then optimized them through random grid search to enhance performance in terms of accuracy and computational efficiency. The models were trained using either SchNet or PaiNN representations, each with a radial cutoff of 10 atomic units (corresponding to 5.3 Å). The network architecture for learning the representation included 3 interaction layers for PaiNN-based models and 6 interaction layers for SchNet-based models. In all models, 256 features were employed to represent the atoms of a molecule, while three layers were utilized in each property network. The initial learning rate was set to 1×10^{-4} and was decreased by a factor of 0.8 after 50 epochs without improvement in the loss function. The numbers of training, test, and validation points for ethene (C_2H_4), propene (C_3H_6), butene (C_4H_8) and methylenimmonium cation ($CH_2NH_2^+$) are compiled in Table S3.

Table S3 Absolute number (and relative ratio) of training, validation and test points employed in the training of SpaiNN models for the indicated molecules.

**molecule**	training	**validation**	test
**ethene** (C_2H_4)	4,500 (0.75)	**500 (0.08)**	1,000 (0.17)
**propene** (C_3H_6)	4,500 (0.75)	**500 (0.08)**	1,000 (0.17)
**butene** (C_4H_8)	10,000 (0.77)	**2,000 (0.15)**	1,000 (0.08)
**methylenimmonium cation** ($CH_2NH_2^+$)	2,500 (0.63)	**250 (0.06)**	1,250 (0.31)

S3.2 Performance: Training on XAlkeneDB

Table S4 Mean absolute errors (MAEs) and root mean squared errors (RMSEs) of SpaiNN models for ethylene, propylene, and butene using SchNet or PaiNN representation, respectively. The respective models were trained on the energies (E_j) and forces ($\mathbf{F}_j$) of the three lowest singlet states (S_0, S_1, and S_2) and the non-adiabatic coupling vectors ($\mathbf{C}_{ij}$) between these states. The errors are reported for each individual state or coupling and the averaged errors. The errors of PaiNN-based models are highlighted in gray and the lowest error when comparing PaiNN- and SchNet-based models are indicated by bold numbers. To account for trade-offs between the target properties, a weight-factor of 1.0 was employed for energies, forces and non-adiabatic couplings, while a factor of 0.1 was used for the dipoles.

Property	MAE (RMSE)					
	PaiNN			SchNet		
	C_2H_4	C_3H_6	C_4H_8	C_2H_4	C_3H_6	C_4H_8
E_j in eV	0.004 (0.01)	**0.054 (0.08)**	**0.002 (0.01)**	**0.003 (0.01)**	0.065 (0.11)	0.011 (0.02)
E_0	0.003 (0.01)	0.022 (0.03)	0.002 (0.01)	0.005 (0.01)	0.016 (0.02)	0.003 (0.01)
E_1	0.004 (0.01)	0.035 (0.04)	0.001 (0.01)	0.003 (0.01)	0.061 (0.06)	0.007 (0.01)
E_2	0.005 (0.01)	0.036 (0.05)	0.001 (0.01)	0.001 (0.01)	0.023 (0.02)	0.010 (0.01)
$\mathbf{F}_j$ in eV·Å^{-1}	**0.020 (0.03)**	**0.291 (0.96)**	**0.014 (0.02)**	0.022 (0.05)	0.409 (0.79)	0.062 (0.09)
$\mathbf{F}_0$	0.042 (0.06)	0.137 (0.18)	0.011 (0.01)	0.067 (0.10)	0.323 (0.50)	0.037 (0.05)
$\mathbf{F}_1$	0.033 (0.05)	0.171 (0.25)	0.013 (0.02)	0.019 (0.03)	0.276 (0.36)	0.045 (0.06)
$\mathbf{F}_2$	0.015 (0.02)	0.368 (0.52)	0.010 (0.01)	0.016 (0.02)	0.384 (0.56)	0.043 (0.06)
$\mathbf{C}_{ij}$	**0.024 (0.08)**	**0.021 (0.04)**	**0.004 (0.01)**	0.034 (0.07)	0.031 (0.05)	0.024 (0.06)
$\mathbf{C}_{01}$	0.008 (0.02)	0.012 (0.02)	0.001 (0.01)	0.032 (0.04)	0.026 (0.04)	0.010 (0.01)
$\mathbf{C}_{02}$	0.004 (0.01)	0.014 (0.02)	0.002 (0.01)	0.020 (0.04)	0.021 (0.03)	0.011 (0.02)
$\mathbf{C}_{12}$	0.006 (0.01)	0.019 (0.03)	0.001 (0.01)	0.021 (0.03)	0.056 (0.09)	0.011 (0.02)

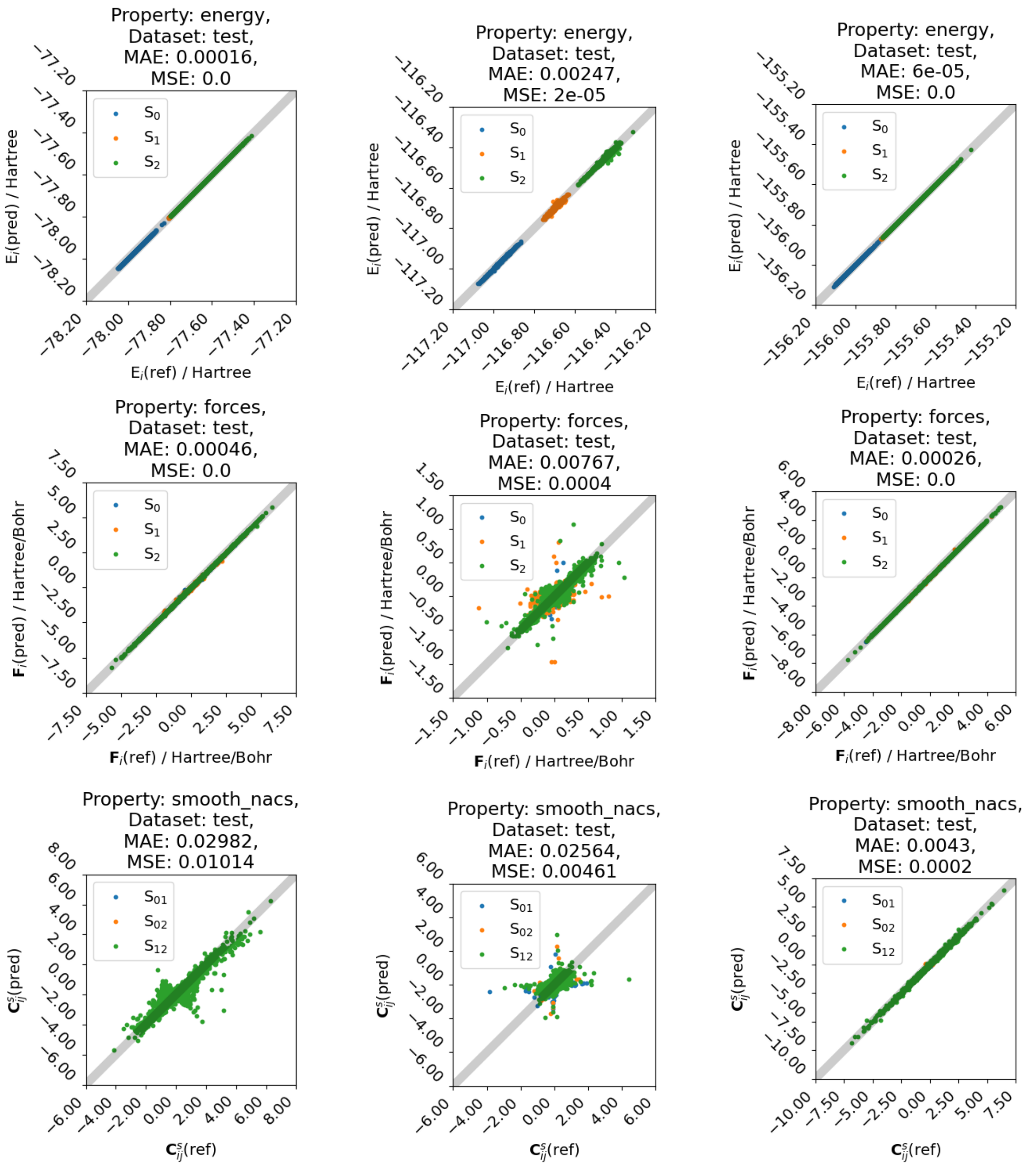

(i) C_2H_4 **(ii)** C_3H_6 **(iii)** C_4H_8

Fig. S4 Scatter plots showing the distribution of the energies (top), forces (middle) and non-adiabatic coupling vectors (bottom) obtained from SpaiNN models trained on these three properties for the lowest three singlet states of ethene (i), propene (ii), and butene (iii) using PaiNN representation. The plots were generated using the `spainn.plotting` module of the `spainn` package.

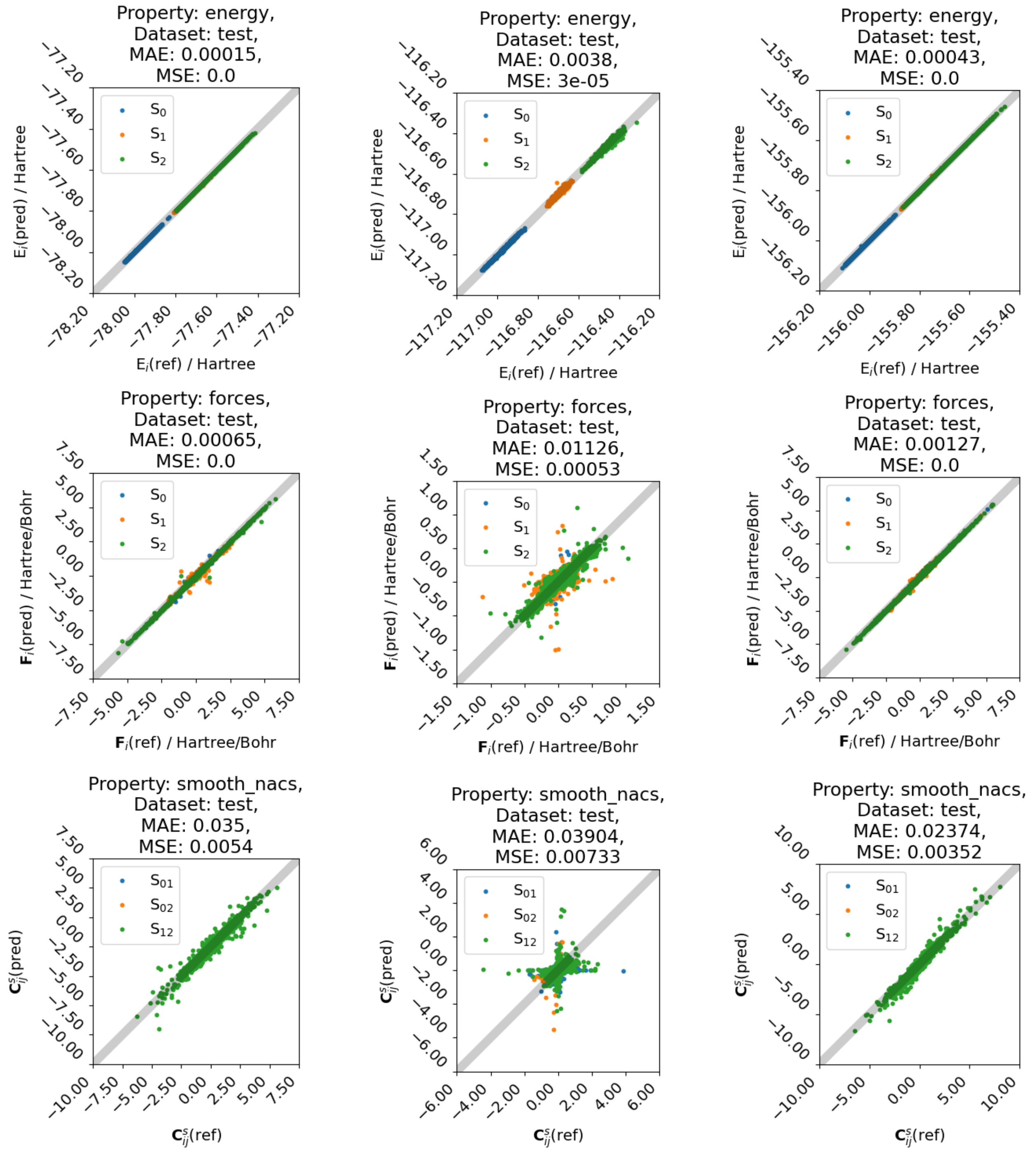

(i) C_2H_4 **(ii)** C_3H_6 **(iii)** C_4H_8

Fig. S5 Scatter plots showing the distribution of the energies (top), forces (middle) and non-adiabatic coupling vectors (bottom) obtained from SpaiNN models trained on these three properties for the lowest three singlet states of ethene (i), propene (ii), and butene (iii) using SchNet representation. The plots were generated using the `spainn.plotting` module of the `spainn` package.

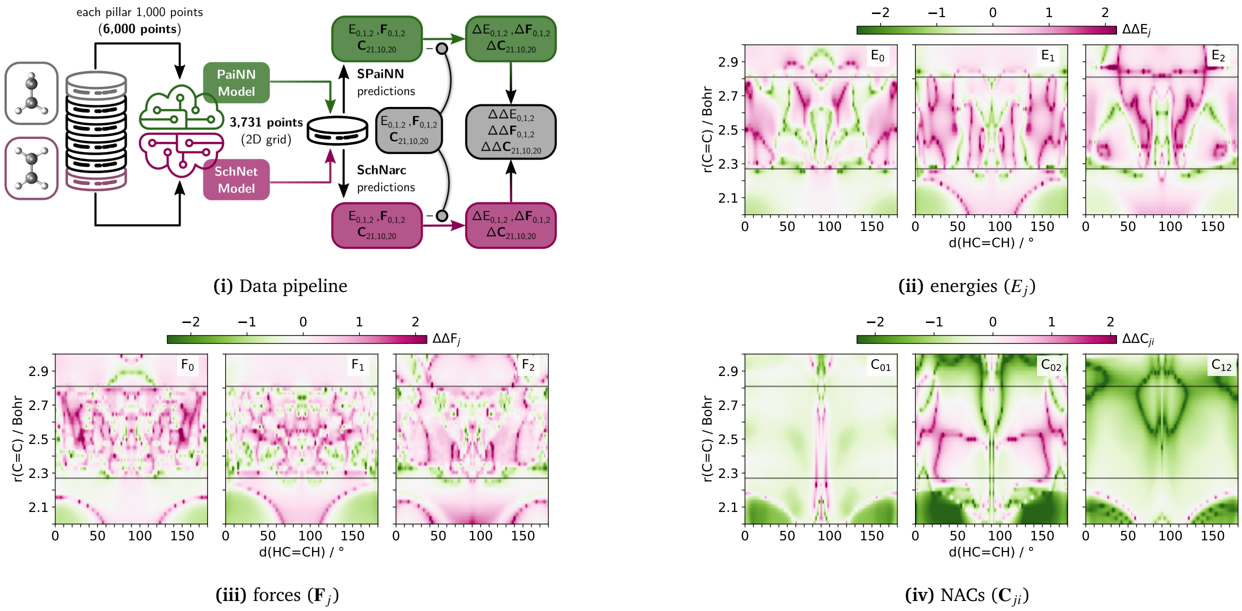

(i) Data pipeline **(ii)** energies (E_j)

(iii) forces ($\mathbf{F}_j$) **(iv)** NACs ($\mathbf{C}_{ji}$)

Fig. S6 Error-difference plots depicting potential energies (ii), forces (iii), and NACs (iv) for ethene (C_2H_4), as predicted by PaiNN- and SchNet-based SpaiNN models, over a 2D grid formed by sampling the C=C alkene bond length and HC=CH dihedral angle (bond rotation). The color scheme denotes superior performance of PaiNN-based models in green and SchNet-based models in pink. Notably, none of the data points used in grid calculation were part of the training dataset. The process for generating these error-difference plots is depicted in the top left panel (i).

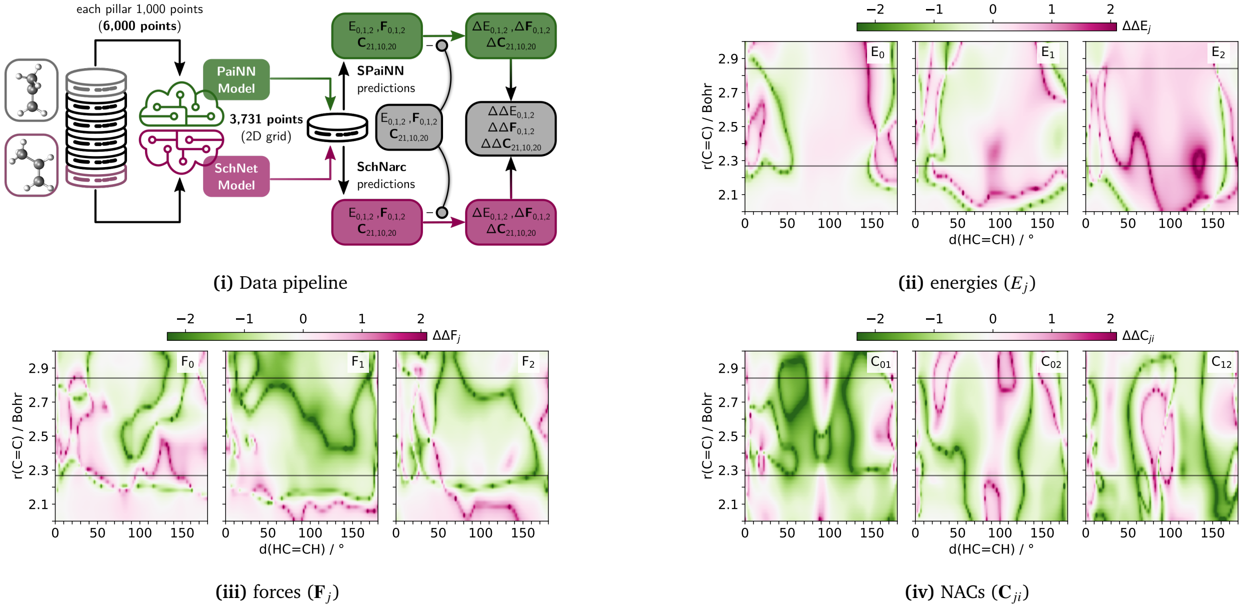

(i) Data pipeline **(ii)** energies (E_j)

(iii) forces ($\mathbf{F}_j$) **(iv)** NACs ($\mathbf{C}_{ji}$)

Fig. S7 Error-difference plots depicting potential energies (ii), forces (iii), and NACs (iv) for propene (C_3H_6), as predicted by PaiNN- and SchNet-based SpaiNN models, over a 2D grid formed by sampling the C=C alkene bond length and HC=CH dihedral angle (bond rotation). The color scheme denotes superior performance of PaiNN-based models in green and SchNet-based models in pink. Notably, none of the data points used in grid calculation were part of the training dataset. The process for generating these error-difference plots is depicted in the top left panel (i).

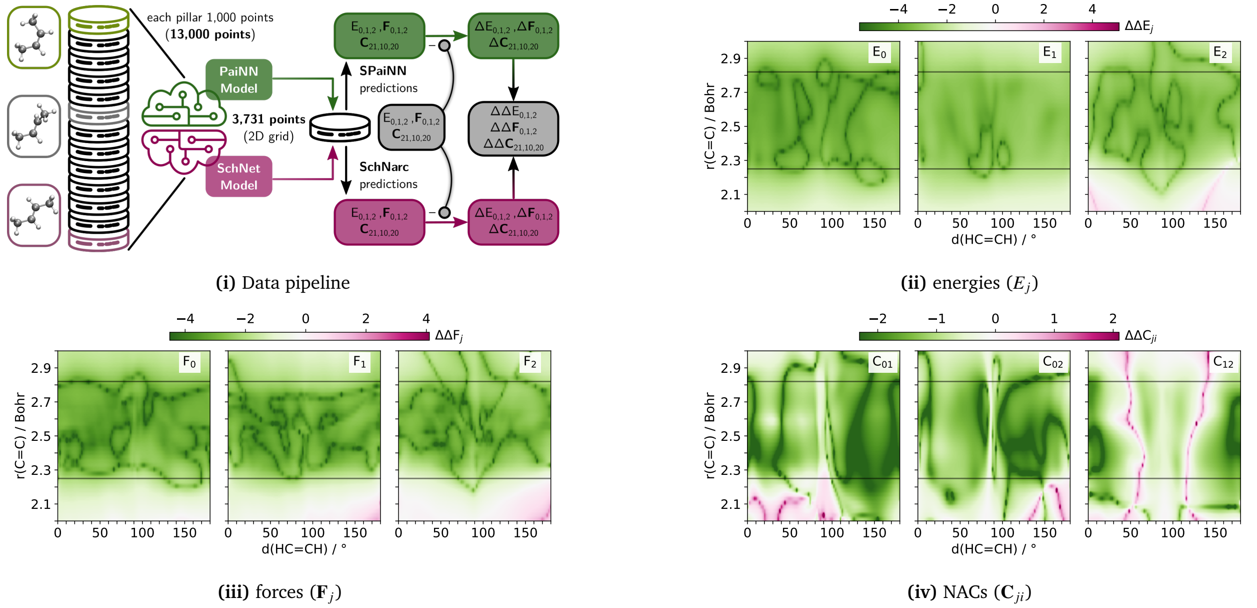

(i) Data pipeline

(ii) energies (E_j)

(iii) forces ($\mathbf{F}_j$)

(iv) NACs ($\mathbf{C}_{ji}$)

Fig. S8 Error-difference plots depicting potential energies (ii), forces (iii), and NACs (iv) for butene (C_4H_8), as predicted by PaiNN- and SchNet-based SpaiNN models, over a 2D grid formed by sampling the C=C alkene bond length and HC=CH dihedral angle (bond rotation). The color scheme denotes superior performance of PaiNN-based models in green and SchNet-based models in pink. Notably, none of the data points used in grid calculation were part of the training dataset. The process for generating these error-difference plots is depicted in the top left panel (i).

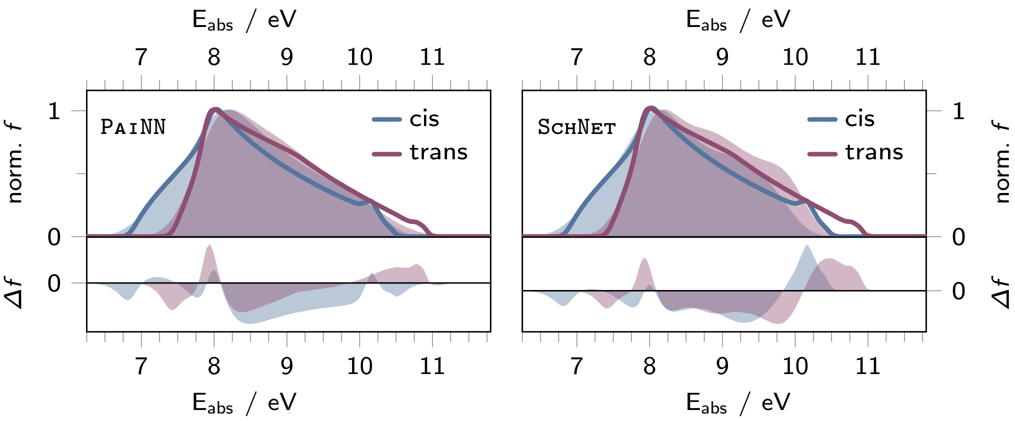

Fig. S9 Simulated absorption spectra depicting S_0 to S_1 excitation in both *cis*- (blue, a) and *trans*-butene (purple, a). Reference spectra, acquired for 451 geometries of *cis*- and *trans*-butene at SA(3)-CASSCF(2,2)/cc-pVDZ[1] level of theory, are represented as solid, unfilled lines. The filled curves display the absorption spectra derived from SpaiNN models using PaiNN (left) or SchNet (right) representation for the respective structures. Simulated/predicted vertical excitation energies and corresponding oscillator strengths were spectrally broadened using Gaussian functions with a full width at half maximum of 0.1 eV.

S3.3 Performance: Training on $CH_2NH_2^+$ database

To assess the accuracy of the models, scatter plots for energies, forces, NACs, and dipole moment vectors are shown in Figure S10 for SPaiNN models employing PaiNN and SchNet representation, respectively. Colors

Table S5 Mean absolute error (MAE) and root mean squared error (RMSE) of the trained properties (E_j, $\mathbf{F}_{ji}$, $\mathbf{C}_{ji}$, and μ_{ji}) of models trained for the methylenimmonium cation using SchNet and PaiNN representation. t indicates the weight-factor used for every property during training (*cf.* Eq. 4).

Property / Unit	PaiNN MAE (RMSE)	SchNet MAE (RMSE)	t
Energies / eV	0.055 (0.11)	0.091 (0.16)	1.0
Forces / eV·Å^{-1}	0.280 (0.57)	0.407 (0.74)	1.0
NACs	0.179 (3.10)	0.233 (3.15)	1.0
Dipoles / D	0.087 (0.19)	0.125 (0.28)	0.1

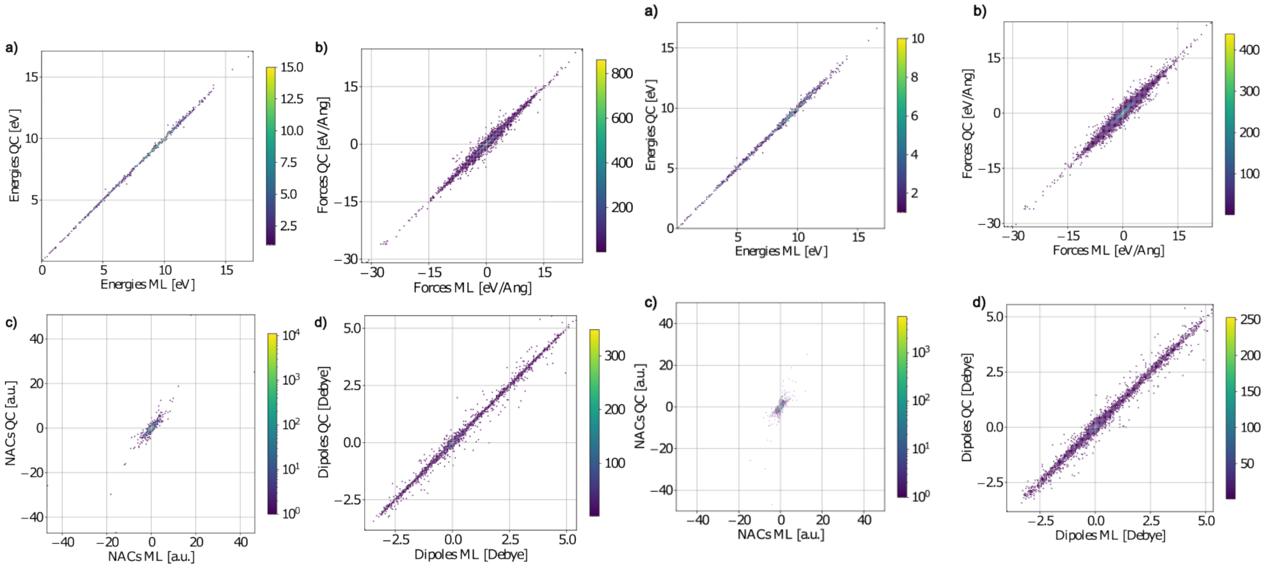

(i) PaiNN representation **(ii)** SchNet representation

Fig. S10 Scatter plots showing the distribution of the energies (a), forces (b), non-adiabatic coupling vectors (c), and dipole moment vectors (d) obtained from SPaiNN models that are trained on these four properties for the lowest three singlet states of the methylenimmonium cation ($CH_2NH_2^+$) using PaiNN (left) or SchNet representation (right).

in the scatter plots indicate the amount of data points that lie within a certain region. As it is visible, SPaiNN models outperform SchNarc models in terms of accuracy for all fitted properties. This is especially true for NACs, where SchNarc predicts the maximum to be around ±10 a.u., while the highest NAC values fall within the range of ±30 a.u., which is much better captured by SPaiNN (*cf.* MAE plots in Figure S10(i) *vs.* (ii)).

Finally, an example of a NAC component along the C-N rotation is shown in Figure S13. NACs are scaled for better visibility such that the largest component obtained from QC is at 1. As can be seen, at the regions of conical intersections, SPaiNN captures the magnitude of the NACs almost exactly compared to the reference method, while SchNarc overestimates the value. The potential energy surfaces are also captured more accurately with SPaiNN than SchNarc. However, the NACs close to zero are more accurate in SchNarc than SPaiNN. Noticeably, the NACs show a decrease, when the corresponding energies are close to each other, which might be an effect due to the Berry phase or the Born-Oppenheimer breakdown and is thus considered an artifact. This effect is corrected for with ML models.

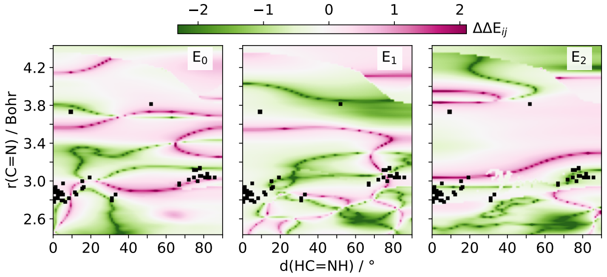

(i) energies (E_j)

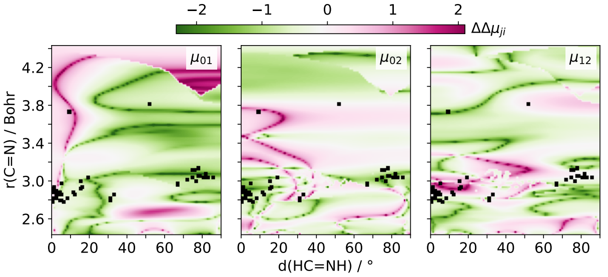

(ii) dipoles (μ_{ji})

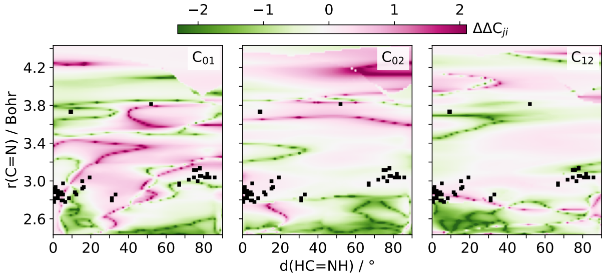

(iii) NACs ($\mathbf{C}_{ji}$)

Fig. S11 Error-difference plots depicting potential energies (i), dipoles (ii), and NACs (iii) for the methylenimmonium cation ($CH_2NH_2^+$), as predicted by PaiNN- and SchNet-based SpaiNN models, over a 2D grid formed by sampling the C=N bond length and HC=NH dihedral angle (bond rotation). The color scheme denotes superior performance of PaiNN-based models in green and SchNet-based models in pink. Notably, none of the data points used in grid calculation were part of the training dataset.

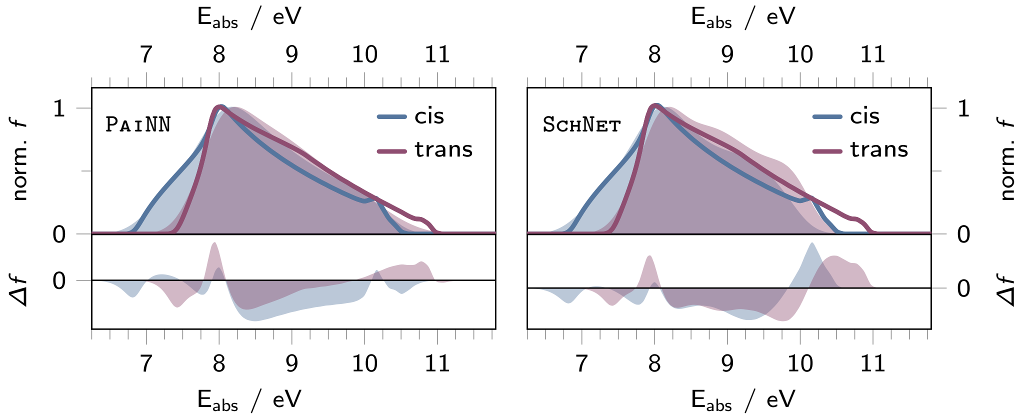

Fig. S12 Simulated absorption spectra of $CH_2NH_2^+$ depicting the sum of the S_0 to S_1 and the S_0 to S_2 excitation. Reference spectra, acquired for 20 k geometries of $CH_2NH_2^+$ at MR-CISD(6,4)/aug-ccpVDZ[27] level of theory, are represented as solid, unfilled lines. The filled curves display the absorption spectra derived from SpaiNN models using PaiNN (left) or SchNet (right) representation for the respective structures. Simulated/predicted vertical excitation energies and corresponding oscillator strengths were spectrally broadened using Gaussian functions with a full width at half maximum of 0.1 eV.

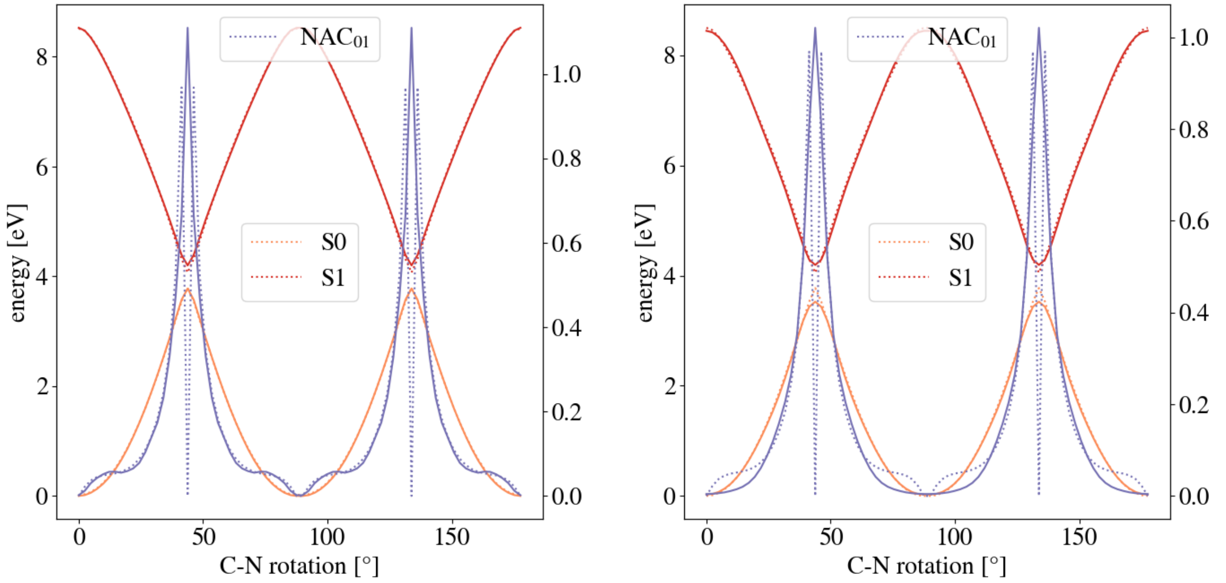

(i) PaiNN

(ii) SchNet

Fig. S13 Potential energy surfaces and NACs (of C atom in z direction as an example) of quantum chemistry (QC) and PaiNN- (i) and SchNet-based (ii) SpaiNN models along the C-N bond rotation for the S_2 and S_1 states. Solid lines represent curves obtained from ML models and dotted lines from QC.

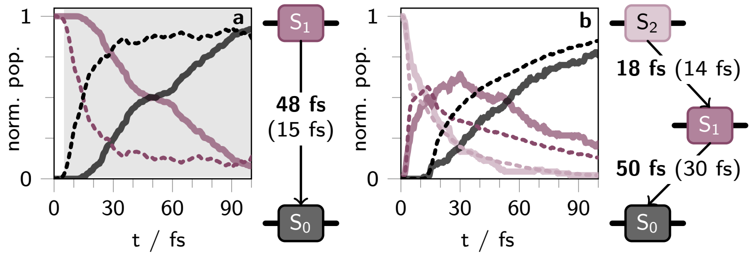

Fig. S14 Temporal evolution of population in the two lowest singlet excited states upon photoexcitation into S_1 of *cis*-butene (a) and the three lowest singlet excited states of $CH_2NH_2^+$ upon excitation into S_2 (b) as derived from SHARC simulations spanning 100 fs (with a time-step of 0.5 fs). The solid lines represent outcomes from 100 SHARC trajectories on SA(3)-CASSCF(2,2)/cc-pVDZ (a) or MR-CISD/aug-cc-pVDZ level of theory, respectively.[1,10] Dashed lines reflect results from 600 (a) or 3846 SHARC trajectories (b), incorporating non-adiabatic couplings, energies, and forces predicted by a SpaiNN model leveraging SchNet representation. The background color is white in regions where SpaiNN models yield reasonable results, as determined by the total energy of the molecules at each time-step, while other regions are shaded gray. Kinetic rate constants for a sequential kinetics scheme were derived using KiMoPack and are presented alongside the population plots. Bold numbers denote the excited-state lifetimes as obtained from reference calculations, the ML lifetimes are given in parenthesis.

Notes and references

1 C. Müller and A. Tkatchenko, *XAlkeneDB: A database illuminating the electronic ground and excited state quantum chemical features of ethene, propene and butene*, 2024, `https://doi.org/10.5281/zenodo.10736050`.

2 S. Mai, P. Marquetand and L. González, *Int. J. Quantum Chem.*, 2015, **115**, 1215–1231.

3 S. Mai, P. Marquetand and L. González, *WIREs Comput. Mol. Sci.*, 2018, **8**, e1370.

4 K. Schütt, O. Unke and M. Gastegger, *Equivariant message passing for the prediction of tensorial properties and molecular spectra*, 2021, `https://proceedings.mlr.press/v139/schutt21a.html`.

5 *SPaiNN*, `https://github.com/CompPhotoChem/SPaiNN`.

6 https://spainn.readthedocs.io/.

7 K. T. Schütt, S. S. P. Hessmann, N. W. A. Gebauer, J. Lederer and M. Gastegger, *Journal of Chemical Physics*, 2023, **158**, 144801–144801.

8 A. Hjorth Larsen, J. Jørgen Mortensen, J. Blomqvist, I. E. Castelli, R. Christensen, M. Dułak, J. Friis, M. N. Groves, B. Hammer, C. Hargus, E. D. Hermes, P. C. Jennings, P. Bjerre Jensen, J. Kermode, J. R. Kitchin, E. Leonhard Kolsbjerg, J. Kubal, K. Kaasbjerg, S. Lysgaard, J. Bergmann Maronsson, T. Maxson, T. Olsen, L. Pastewka, A. Peterson, C. Rostgaard, J. Schiøtz, O. Schütt, M. Strange, K. S. Thygesen, T. Vegge, L. Vilhelmsen, M. Walter, Z. Zeng and K. W. Jacobsen, *J. Phys.: Cond. Matt.*, 2017, **29**, 273002.

9 J. Westermayr, M. Gastegger and P. Marquetand, *J. Phys. Chem. Lett.*, 2020, **11**, 3828–3834.

10 J. Westermayr, M. Gastegger, M. F. S. J. Menger, S. Mai, L. González and P. Marquetand, *Chemical Science*, 2019, **10**, 8100–8107.

11 *SchNarc*, `https://github.com/schnarc/SchNarc.git`.

12 C. Adamo and V. Barone, *The Journal of Chemical Physics*, 1999, **110**, 6158–6170.

13 M. Ernzerhof and G. E. Scuseria, *The Journal of Chemical Physics*, 1999, **110**, 5029–5036.

14 F. Weigend and R. Ahlrichs, *Physical Chemistry Chemical Physics*, 2005, **7**, 3297.

15 S. Grimme, S. Ehrlich and L. Goerigk, *Journal of Computational Chemistry*, 2011, **32**, 1456–1465.

16 T. Hočevar and J. Demšar, *Journal of Statistical Software*, 2016, **71**, 1–24.

17 S. Grimme, A. Hansen, S. Ehlert and J.-M. Mewes, *The Journal of Chemical Physics*, 2021, **154**, 064103.

18 E. Colomés, Z. Zhan and X. Oriols, *Journal of Computational Electronics*, 2015, **14**, 894–906.

19 M. Hillery, R. F. O'Connell, M. O. Scully and E. P. Wigner, *Physics Reports*, 1984, **106**, 121–167.

20 S. Mai, P. Marquetand and L. González, *WIREs Computational Molecular Science*, 2018, **8**, e1370.

21 S. Mai, D. Avagliano, M. Heindl, P. Marquetand, M. F. S. J. Menger, M. Oppel, F. Plasser, S. Polonius, M. Ruckenbauer, Y. Shu, D. G. Truhlar, L. Zhang, P. Zobel and L. González, *SHARC3.0: Surface Hopping Including Arbitrary Couplings – Program Package for Non-Adiabatic Dynamics*, 2023, `https://zenodo.org/records/7828641`.

22 B. O. Roos, P. R. Taylor and P. E. Siegbahn, *Chem. Phys.*, 1980, **48**, 157–173.

23 B. O. Roos and P. E. M. Siegbahn, *Int. J. Quantum Chem.*, 1980, **17**, 485–500.

24 T. H. Dunning, Jr., *The Journal of Chemical Physics*, 1989, **90**, 1007–1023.

25 F. Aquilante, J. Autschbach, A. Baiardi, S. Battaglia, V. A. Borin, L. F. Chibotaru, I. Conti, L. De Vico, M. Delcey, I. Fdez. Galván, N. Ferré, L. Freitag, M. Garavelli, X. Gong, S. Knecht, E. D. Larsson, R. Lindh, M. Lundberg, P. A. Malmqvist, A. Nenov, J. Norell, M. Odelius, M. Olivucci, T. B. Pedersen, L. Pedraza-González, Q. M. Phung, K. Pierloot, M. Reiher, I. Schapiro, J. Segarra-Martí, F. Segatta, L. Seijo, S. Sen, D.-C. Sergentu, C. J. Stein, L. Ungur, M. Vacher, A. Valentini and V. Veryazov, *The Journal of Chemical Physics*, 2020, **152**, 214117.

26 R. A. Kendall, J. Dunning, Thom H. and R. J. Harrison, *The Journal of Chemical Physics*, 1992, **96**, 6796–

6806.
27 J. Westermayr and P. Marquetand, *J. Chem. Phys*, 2020, **153**, 154112.
